# Joint effects of severe obesity and inflammation on mortality in critically ill non−ST−segment elevation myocardial infarction patients: a cohort study with external validation

**DOI:** 10.3389/fendo.2026.1850802

**Published:** 2026-06-24

**Authors:** Yuqing Li, Yuhang Wang, Pengju Lu, Jiaxin Wang, Weiwei Tian, Ran Chu, Jingxi Chen, Lai Jiang, Changping Li, Yin Liu, Jing Gao

**Affiliations:** 1 Clinical School of Thoracic, Tianjin Medical University, Tianjin, China; 2 Department of Cardiology, Tianjin Chest Hospital, Tianjin, China; 3 Chest Hospital, Tianjin University, Tianjin, China; 4 School of Public Health, Tianjin Medical University, Tianjin, China; 5 Cardiovascular Institute, Tianjin Chest Hospital, Tianjin, China

**Keywords:** all-cause mortality, body mass index, coronary care unit, C-reactive protein, non-ST-segment elevation myocardial infarction

## Abstract

**Background:**

To investigate the association between body mass index (BMI) and both in-hospital and 10-year all-cause mortality in critically ill non−ST−segment elevation myocardial infarction (NSTEMI) patients, and to evaluate the incremental value of C-reactive protein (CRP) in risk stratification.

**Methods:**

This multinational study included 7,815 critically ill NSTEMI patients from three cohorts. We first analyzed an original discovery cohort (TAMI, n = 5,010) from a Chinese tertiary hospital, then externally validated the findings in two independent US cohorts (MIMIC-IV, n = 1,208; eICU-CRD, n = 1,597). BMI was categorized according to WHO criteria, and CRP was dichotomized at 2 mg/L. Multivariable Cox regression and restricted cubic splines were used to assess mortality risks.

**Results:**

In the TAMI cohort, severe obesity (BMI ≥ 35 kg/m^2^) was independently associated with increased risks of in-hospital (HR 1.69; 95% CI: 1.08-2.65; *p* = 0.022) and 10-year (HR 1.68; 95% CI: 1.31-2.15; *p* < 0.001) all-cause mortality. Overweight and obesity I were associated with lower mortality risk compared with normal weight. The absolute in-hospital mortality rate was 8.36% in the severe obesity group, compared with 1.55% in the overweight group. The coexistence of severe obesity and elevated CRP (≥ 2 mg/L) identified a high-risk clinical profile, with no significant interaction between these two factors for either outcome (*p* for interaction = 0.178 for in-hospital and 0.169 for 10-year all-cause mortality). Adding CRP to the base model (Model 3) significantly improved risk prediction, with an increase in AUC from 0.764 to 0.768 (*p* = 0.021) and significant improvements in reclassification and discrimination metrics (all *p* < 0.05). The pooled meta-analysis results across the three databases were consistent.

**Conclusions:**

BMI exhibits a U-shaped association with mortality in critically ill NSTEMI patients. Severe obesity combined with elevated inflammation identifies a high-risk clinical profile, supporting integrated metabolic-inflammatory risk stratification.

## Graphical Abstract

**Figure:**
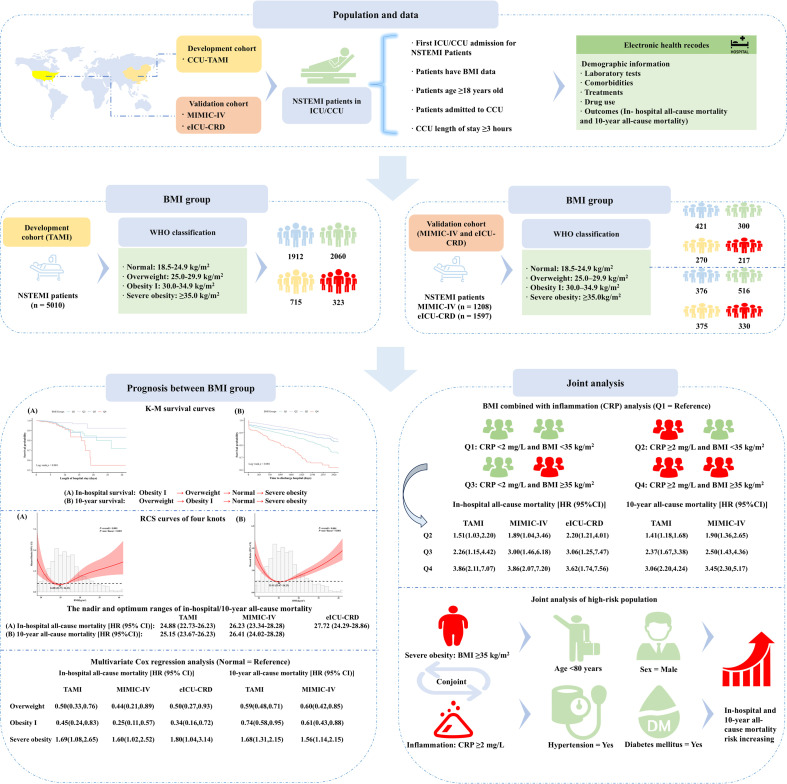
BMI, body mass index; NSTEMI, non-ST-segment elevation myocardial infarction; CCU, coronary care unit; ICU, intensive care unit; TAMI, Tianjin Inpatient Acute Myocardial Infarction; MIMIC-IV, Medical Information Mart for Intensive Care IV; eICU-CRD, eICU Collaborative Research Database; CRP, C-reactive protein; K-M, Kaplan-Meier; RCS, restricted cubic spline; HR, hazard ratio; CI, confidence interval. Values in parentheses indicate 95% CI.

## 1 Introduction

The escalating prevalence of obesity constitutes a major global health challenge. According to the Global Burden of Disease (GBD) Study, approximately half of all adults are living with obesity, as are over one-fifth of children and adolescents worldwide (1). However, in 2015, elevated body mass index (BMI) was responsible for 4 million deaths globally, with more than half attributable to cardiovascular diseases (CVD) (2). Obesity unequivocally increases the risk of acute myocardial infarction (AMI) and premature mortality (3). Moreover, obese AMI patients exhibit a more pronounced inflammatory state than their normal-weight counterparts (4).

In the United States, nearly half of all patients with AMI are admitted to the intensive care unit (ICU), with mortality rates ranging from 14% to 50% (5). Paradoxically, although obesity increases the risk of AMI, it has been associated with improved in-hospital survival in various critical conditions, including heart failure (HF), acute kidney injury (AKI), type 2 diabetes mellitus (DM), and sepsis, a phenomenon often termed the “obesity paradox” (6–8).

The prognostic implication of obesity in critically ill patients with non-ST-segment elevation myocardial infarction (NSTEMI). However, remains less clear and warrants specific investigation. Unlike ST-segment elevation myocardial infarction (STEMI), which is characterized by complete coronary occlusion mandating immediate revascularization, NSTEMI often involves subtotal occlusion, presents with greater clinical heterogeneity, and necessitates management focused on longitudinal risk stratification rather than time-dependent reperfusion (9). Consequently, identifying modifiable risk factors, such as BMI, for predicting mortality in critically ill NSTEMI patients admitted to the coronary care unit (CCU), an exceptionally high-risk cohort, is paramount for refining risk stratification and personalizing management (10).

C-reactive protein (CRP), a classic acute-phase reactant, is a well-established biomarker of systemic inflammation and a robust predictor of future cardiovascular events (11). Recent meta-analyses suggest that baseline CRP levels may modulate the long-term benefit of lipid-lowering therapies in AMI patients (12). Furthermore, in AMI complicated by cardiogenic shock, elevated CRP is associated with increased 30-day all-cause mortality (13). However, evidence evaluating the prognostic value of acute-phase CRP specifically in NSTEMI remains scarce, typically derived from studies with small sample sizes, short-term follow-up, or undifferentiated AMI populations (14).

Therefore, leveraging an original institutional cohort (the Chinese Tianjin Inpatient Acute Myocardial Infarction [TAMI] registry) and two independent external validation cohorts (the Medical Information Mart for Intensive Care IV [MIMIC-IV] and eICU Collaborative Research Database [eICU-CRD]), this study aimed to investigate the association between BMI and both in-hospital and 10-year all-cause mortality in critically ill NSTEMI patients admitted to the CCU. Additionally, we sought to evaluate the incremental prognostic value of CRP to provide a scientific rationale for integrated weight and inflammatory management in this vulnerable population (11).

## 2 Materials and methods

### 2.1 Study design and participants

We conducted a retrospective, multicohort study using an original institutional cohort (the TAMI registry from Tianjin Chest Hospital, China) and two independent external validation cohorts (the MIMIC-IV and eICU-CRD databases from the United States). Detailed information is provided in **Supplementary Table 1**. Previous studies support the comparability and analytical consistency across these databases (15).

Data extraction was performed by Yuqing Li, who completed the relevant Collaborative Institutional Training Initiative (CITI) program (ID: 13947201) and holds certified access to the databases.

NSTEMI diagnosis adhered to the Fourth Universal Definition of Myocardial Infarction (16), with cohort-specific criteria detailed in **Supplementary Table 2**.

**Figure 1:**
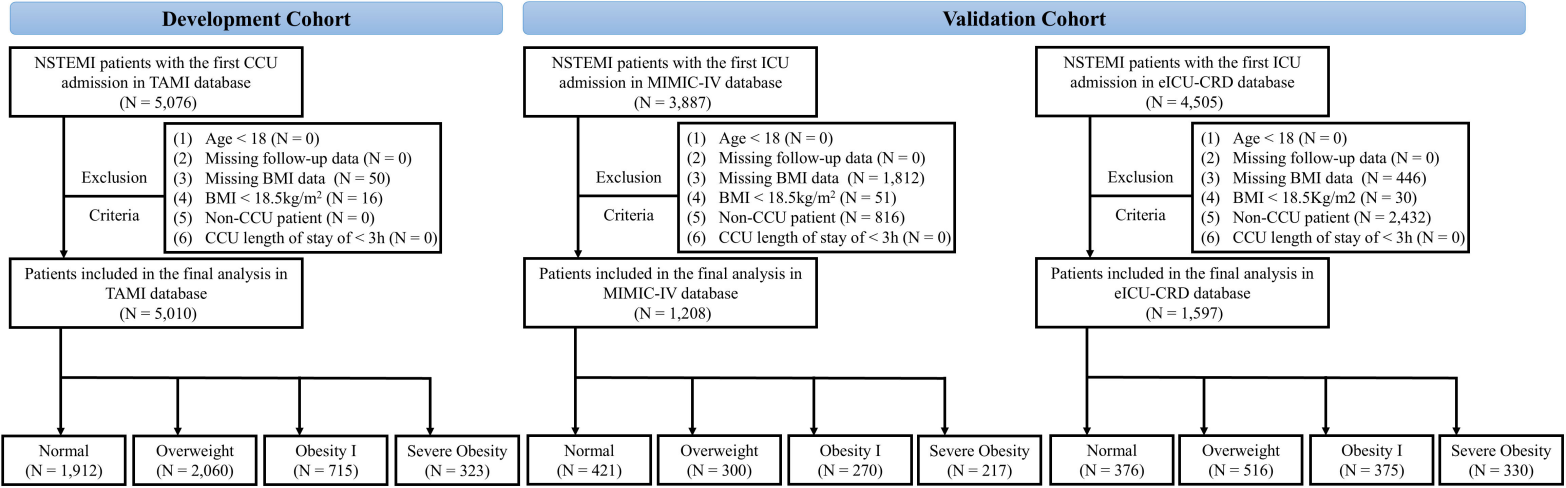
Research flow chart. NSTEMI, non-ST-segment elevation myocardial infarction; CCU, coronary care unit; BMI, body mass index; ICU, intensive care unit; TAMI, Tianjin Inpatient Acute Myocardial Infarction; MIMIC-IV, Medical Information Mart for Intensive Care IV; eICU-CRD, eICU Collaborative Research Database.

We included all patients with a first-time ICU/CCU admission for NSTEMI. Exclusion criteria were: (1) age <18 years; (2) missing follow-up data; (3) missing BMI data; (4) BMI <18.5 kg/m^2^; (5) admission to a non-CCU; and (6) length of stay in CCU < 3 hours. The flow of patient selection is illustrated in **Figure 1**. The final analysis included 5,010 patients from the TAMI cohort, and 1,208 and 1,597 patients from the MIMIC-IV and eICU-CRD cohorts, respectively.

This study was approved by the Ethics Committee of Tianjin Chest Hospital (Ethical number: 2018KY-010-01). All participating institutions obtained approval from their respective ethics committees, and the study was conducted in strict accordance with the principles of the Declaration of Helsinki.

### 2.2 Data collection

Extracted variables included: (1) Anthropometric and demographics: age, sex, height, weight, and BMI; (2) Comorbidities: hypertension (HBP), DM, hyperlipidemia (HL), atrial fibrillation (AF), and stroke; (3) laboratory parameters: cardiac troponin T (cTnT), creatine kinase (CK), creatine kinase-MB (CK-MB), CRP, white blood cell (WBC), creatinine, urea nitrogen (UN), low-density lipoprotein cholesterol (LDL-C), high-density lipoprotein cholesterol (HDL-C) and albumin; (4) Treatments: ventilator, intra-aortic balloon pump (IABP), percutaneous coronary intervention (PCI), and coronary artery bypass grafting (CABG); and (5) Medications use: aspirin, clopidogrel, statins, β-blockers, anticoagulants, angiotensin-converting enzyme inhibitors (ACEI)/angiotensin receptor blockers (ARB). For all laboratory measurements, we extracted the first recorded value after CCU admission.

We acknowledge that the timing of these measurements varied between days 1 and 3 post-admission, reflecting real-world clinical practice rather than a strictly standardized protocol. However, the first measured value is the earliest available clinical indicator available to attending physicians and has been shown in numerous studies to carry independent prognostic significance for short- and long-term outcomes in acute coronary syndromes (17, 18). While repeated measurements might provide additional dynamic information, the first measured, being the most clinically actionable time point, supports its validity in outcome prediction.

In the TAMI cohort, in-hospital data were extracted from the hospital information system (HIS) by certified cardiologists. Self-reported information was verified through face-to-face interviews with medical staff to ensure data authenticity and accuracy. Data from the MIMIC-IV (version 3.0) and eICU-CRD (version 2.0) cohorts were extracted using structured query language (SQL) and PostgreSQL (version 16.0). The data query and extraction code are publicly available from the websites (https://github.com/MIT-LCP/mimic-code and https://physionet.org/content/eicu-crd/2.0/) (19, 20).

### 2.3 Definitions of variables

For the primary analysis, participants in all three cohorts were classified according to WHO criteria into four groups: normal, overweight, obesity I, and severe obesity (21). Specific BMI ranges were defined as follows: normal weight (18.5-24.9 kg/m^2^), overweight (25.0-29.9 kg/m^2^), obesity I (30.0-34.9 kg/m^2^), and severe obesity (≥ 35.0 kg/m^2^). Patients with BMI < 18.5 kg/m^2^ (underweight) were excluded from the study as per the exclusion criteria. Specific cut-off values are detailed in **Supplementary Table 3**. The Chinese obesity classification criteria were applied to the TAMI cohort exclusively as a prespecified sensitivity analysis.

CRP was used as a surrogate marker of systemic inflammation. Patients were dichotomized into CRP < 2 mg/L and ≥ 2 mg/L groups. The choice of 2 mg/L is not arbitrary but is based on established clinical and epidemiological evidence. Firstly, the concept of residual inflammatory risk has been well validated in patients with atherosclerotic CVD, where a CRP level ≥ 2 mg/L despite optimal statin therapy identifies individuals at persistently high risk of recurrent events. This threshold has been used in major trials including JUPITER and CANTOS (22–24). Secondly, research by Gao et al. demonstrated that an on-treatment CRP ≥ 2 mg/L is associated with significantly higher cardiovascular mortality, even after LDL-C lowering (25). The same threshold has been recommended in European consensus documents to define “high inflammatory risk” in secondary prevention (26). Restricted cubic spline (RCS) analysis with four knots was performed in the TAMI cohort using CRP, adjusted for Model 3 covariates. Nonlinearity was tested. The identified threshold was applied to the MIMIC-IV and eICU-CRD cohorts. Results are shown in **Supplementary Figure 1**. Finally, we chose 2 mg/L *a priori* as a clinically interpretable and externally reproducible threshold, rather than deriving it from our dataset to avoid overfitting, ensuring that our findings can be directly compared with other studies and guidelines (27).

### 2.4 Follow-up and outcomes

The primary endpoints were in-hospital and 10-year all-cause mortality (death from any cause) (28). Follow-up time was determined as shown in **Supplementary Figure 2**.

### 2.5 Statistical analysis

Patients were stratified by BMI category. Continuous variables with normal distribution are presented as mean ± standard deviation and compared using Student’s t-test. Non-normally distributed continuous variables are presented as median (interquartile range) and compared using the Kruskal-Wallis test. Categorical variables are presented as counts and percentages and compared using the chi-square test. Due to the lack of data on long-term changes in BMI, we limited the observation period to 10 years after the onset of NSTEMI. Patients with more than 10 years of follow-up were censored at 10 years.

Differences in survival probability across BMI groups were assessed using the log-rank test and visualized with Kaplan-Meier (K-M) curves. The association between BMI categories and all-cause mortality was evaluated using multivariable Cox proportional hazards models. To guide variable selection, we constructed a directed acyclic graph (DAG) to formalize our causal framework (**Supplementary Figure 3**). The DAG identified age, sex, HBP, DM, HL, stroke, and AF as the minimal sufficient adjustment set. Treatments and medications were considered mediators and therefore not adjusted for in the primary analysis to avoid overadjustment bias. The proportional hazards assumption was tested using Schoenfeld residual tests, and no violation was detected (global *p* > 0.05 for all models; **Supplementary Table 4**). Three sequential models were constructed: Model 1: unadjusted; Model 2: adjusted for age and sex; Model 3: further adjusted for HBP, DM, HL, stroke, and AF.

A four-knot RCS analysis was performed based on the covariates in Model 3. Knots were placed at the 5th, 35th, 65th, and 95th percentiles of the BMI distribution, as recommended by Harrell for an optimal balance between flexibility and overfitting (29). Variance inflation factors (VIF) were calculated for all covariates to assess multicollinearity. As all VIFs were < 2 (**Supplementary Table 5**), significant collinearity was deemed absent.

Stratified analyses were performed by age, sex, HBP, and DM status to assess effect modification. To further delineate high-risk populations, patients were cross-classified into four groups based on severe obesity (BMI ≥ 35 kg/m^2^) and CRP (2 mg/L): low BMI/low CRP (BMI < 35 kg/m^2^ and CRP < 2 mg/L), low BMI/high CRP (BMI < 35 kg/m^2^ and CRP ≥ 2 mg/L), high BMI/low CRP (BMI ≥ 35 kg/m^2^ and CRP < 2 mg/L) and high BMI/high CRP (BMI ≥ 35 kg/m^2^ and CRP ≥ 2 mg/L). Multivariable Cox regression was used to assess mortality risk across these groups. To evaluate the consistency of our findings, we presented cohort-specific baseline characteristics and performed formal interaction tests. Product terms between cohort and exposure (severe obesity; elevated CRP) were included in fully adjusted Cox models.

The proportion of missing data for key variables is summarized in **Supplementary Table 6**. Variables with missingness included CRP, cTnT, creatinine, and others. Missing values were handled using multiple imputation by chained equations (MICE), generating five imputed datasets with results pooled using Rubin’s rules (30).

To evaluate the interaction between severe obesity and elevated CRP, we performed both multiplicative and additive interaction analyses. Multiplicative interaction was tested by including a product term (severe obesity × elevated CRP) in the fully adjusted Cox model. Additive interaction was assessed using the relative excess risk due to interaction (RERI), attributable proportion (AP), and synergy index (SI). The incremental prognostic value of CRP was quantified by changes in the area under the curve (AUC), net reclassification improvement (NRI), and integrated discrimination improvement (IDI). Calibration curves were generated using the rms R package with 1,000 bootstrap resamples. The base model (Model 3) and the incremental model (Model 3 + CRP) were compared. Good calibration was defined as slope close to 1.0 and Emax < 0.1.

Causal mediation analysis was performed to examine whether CRP mediated the association between severe obesity and all-cause mortality. The total effect, direct effect, and indirect effect were estimated using 1,000 bootstrap resamples. Outcome models were adjusted for covariates in Model 3 and included an exposure-mediator interaction term.

Eight sensitivity analyses were conducted to test robustness. First, Fine-Gray competing risk regression was performed treating in-hospital death as a competing event for 10-year mortality. Second, to mitigate reverse causation, patients who died within the first year were excluded. Third, we additionally adjusted for BMI as a continuous variable to test the independent predictive value of BMI categories. Fourth, E-values were calculated to quantify the potential impact of unmeasured confounding. Fifth, the Chinese obesity classification criteria were applied to the TAMI cohort. Sixth, a complete-case analysis excluding patients with missing CRP was performed. Seventh, additional covariates were adjusted (include severity of CAD, revascularization, infarct size biomarkers and nutritional status). Finally, a meta-analysis pooling the three cohorts was conducted using random-effects models with Hartung-Knapp adjustment.

A two-sided *p* value < 0.05 was considered statistically significant. All analyses were performed using R software version 4.3.1.

## 3 Results

### 3.1 Baseline characteristics of participants

A total of 7,815 critically ill NSTEMI patients were included from the TAMI (n = 5,010), MIMIC-IV (n = 1,208), and eICU-CRD (n = 1,597) cohorts. The mean age of NSTEMI patients in the TAMI, MIMIC-IV, and eICU-CRD cohorts was 63.63, 69.82, and 66.83 years, respectively. Mean BMI was 27.00, 29.28, and 30.23 kg/m^2^. In-hospital all-cause mortality rates were 2.99%, 7.28%, and 5.57%, while 10-year all-cause mortality rates were 12.67% and 21.52% for TAMI and MIMIC-IV, respectively.

Baseline characteristics stratified by BMI categories are presented in **Table 1** and **Supplementary Tables 7** and **8**. Across all cohorts, individuals in the highest BMI category (severe obesity) were younger and more likely to be female. They had a greater burden of cardiometabolic comorbidities, a more frequent need for acute hemodynamic support, but lower rates of revascularization. They received more intensive guideline-directed medical therapy but exhibited a more pro-inflammatory and adverse metabolic profile.

**Table 1:** Baseline characteristics of all NSTEMI patients with different BMI levels in the TAMI cohort.

Characteristics	Overall (n = 5,010)	Normal (18.5-24.9) (n = 1,912)	Overweight (25.0-29.9) (n = 2,060)	Obesity I (30.0-34.9) (n = 715)	Severe obesity (≥35.0) (n = 323)	p value
Anthropometric and demographic characteristics						
Age, years	63.63 ± 11.31	65.79 ± 10.37	62.83 ± 10.69	60.78 ± 13.25	62.32 ± 13.44	<0.001
Sex, n (%)						<0.001
Female	1,362 (27.19)	577 (30.18)	500 (24.27)	174 (24.34)	111 (34.37)	
Male	3,648 (72.81)	1,335 (69.82)	1,560 (75.73)	541 (75.66)	212 (65.63)	
Weight, kg	77.36 ± 15.46	65.52 ± 8.95	78.13 ± 7.90	92.58 ± 10.40	108.88 ± 16.09	<0.001
Height, cm	169.07 ± 8.30	168.58 ± 8.25	169.38 ± 7.48	169.91 ± 8.65	168.12 ± 11.83	<0.001
BMI, kg/m2	27.00 ± 4.66	23.00 ± 2.17	27.18 ± 1.34	31.98 ± 1.38	38.45 ± 3.52	<0.001
Comorbidities						
Hypertension, n (%)						<0.001
No	1,457 (29.08)	668 (34.94)	569 (27.62)	161 (22.52)	59 (18.27)	
Yes	3,553 (70.92)	1,244 (65.06)	1,491 (72.38)	554 (77.48)	264 (81.73)	
Diabetes mellitus, n (%)						<0.001
No	3,139 (62.65)	1,256 (65.69)	1,283 (62.28)	427 (59.72)	173 (53.56)	
Yes	1,871 (37.35)	656 (34.31)	777 (37.72)	288 (40.28)	150 (46.44)	
Hyperlipidemia, n (%)						<0.001
No	4,190 (83.63)	1,644 (85.98)	1,696 (82.33)	585 (81.82)	265 (82.04)	
Yes	820 (16.37)	268 (14.02)	364 (17.67)	130 (18.18)	58 (17.96)	
Stroke, n (%)						0.214
No	3,953 (78.90)	1,509 (78.92)	1,636 (79.42)	568 (79.44)	240 (74.30)	
Yes	1,057 (21.10)	403 (21.08)	424 (20.58)	147 (20.56)	83 (25.70)	
Atrial fibrillation, n (%)						0.061
No	4,673 (93.27)	1,783 (93.25)	1,938 (94.08)	660 (92.31)	292 (90.40)	
Yes	337 (6.73)	129 (6.75)	122 (5.92)	55 (7.69)	31 (9.60)	
Laboratory parameters						
cTnT, ng/mL	0.35 (0.16,0.78)	0.36 (0.17,0.85)	0.33 (0.15,0.64)	0.37 (0.16,0.90)	0.33 (0.14,1.02)	0.129
CK, U/L	145.00 (86.00,295.75)	145.00 (82.00,282.00)	145.00 (86.00,286.00)	149.00 (89.00,351.00)	145.00 (94.00,317.00)	0.131
CK-MB, U/L	21.00 (15.00,34.00)	21.00 (15.00,34.00)	21.00 (15.00,33.00)	22.00 (16.00,38.00)	21.00 (16.00,36.00)	0.229
CRP, mg/L	2.60 (1.30,6.30)	2.50 (1.20,6.10)	2.60 (1.30,6.20)	2.80 (1.50,8.60)	2.90 (1.30,6.90)	<0.001
WBC, 109/L	7.65 (6.35,9.20)	7.52 (6.09,8.96)	7.65 (6.43,9.15)	7.93 (6.56,9.64)	7.85 (6.65,9.77)	<0.001
Albumin, g/L	42.00 (39.10,44.50)	41.00 (39.50,43.50)	43.00 (41.90,45.80)	42.90 (40.80,44.90)	39.20 (36.30,41.80)	<0.001
Creatinine, umol/L	79.00 (69.00,91.00)	79.00 (68.00,90.00)	79.00 (70.00,91.00)	80.00 (70.00,93.00)	81.00 (73.00,98.00)	<0.001
UN, mmol/L	5.30 (4.50,6.58)	5.30 (4.40,6.40)	5.30 (4.40,6.40)	5.40 (4.50,6.70)	5.50 (4.80,7.10)	<0.001
LDL-C, mmol/L	2.55 (1.95,3.30)	2.50 (1.92,3.22)	2.55 (1.97,3.32)	2.63 (2.03,3.38)	2.69 (1.97,3.53)	<0.001
HDL-C, mmol/L	1.01 (0.83,1.22)	1.04 (0.86,1.26)	0.99 (0.83,1.19)	0.97 (0.80,1.20)	0.99 (0.82,1.17)	<0.001
Treatment						
Ventilator, n (%)						0.361
No	4,878 (97.37)	1,857 (97.12)	2,014 (97.77)	696 (97.34)	311 (96.28)	
Yes	132 (2.63)	55 (2.88)	46 (2.23)	19 (2.66)	12 (3.72)	
Intra-aortic balloon pump, n (%)						0.483
No	4,970 (99.20)	1,899 (99.32)	2,045 (99.30)	706 (98.74)	320 (99.07)	
Yes	40 (0.80)	13 (0.68)	15 (0.73)	9 (1.26)	3 (0.93)	
Percutaneous coronary intervention, n (%)						<0.001
No	1,919 (38.30)	826 (43.20)	696 (33.79)	246 (34.41)	151 (46.75)	
Yes	3,091 (61.70)	1,086 (56.80)	1,364 (66.21)	469 (65.59)	172 (53.25)	
Coronary artery bypass grafting, n (%)						0.014
No	4,587 (91.56)	1,725 (90.22)	1,889 (91.70)	673 (94.13)	300 (92.88)	
Yes	423 (8.44)	187 (9.78)	171 (8.30)	42 (5.87)	23 (7.12)	
Medication use						
Aspirin, n (%)						<0.001
No	529 (10.56)	240 (12.55)	187 (9.08)	65 (9.09)	37 (11.46)	
Yes	4,481 (89.44)	1,672 (87.45)	1,873 (90.92)	650 (90.91)	286 (88.54)	
Clopidogrel, n (%)						<0.001
No	1,592 (31.78)	680 (35.56)	620 (30.10)	204 (28.53)	88 (27.24)	
Yes	3,418 (68.22)	1,232 (64.44)	1,440 (69.90)	511 (71.47)	235 (72.76)	
Statins, n (%)						0.218
No	444 (8.86)	190 (9.94)	171 (8.30)	58 (8.11)	25 (7.74)	
Yes	4,566 (91.14)	1,722 (90.06)	1,889 (91.70)	657 (91.89)	298 (92.26)	
β-Blockers, n (%)						<0.001
No	1,604 (32.02)	691 (36.14)	635 (30.83)	184 (25.73)	94 (29.10)	
Yes	3,406 (67.98)	1,221 (63.86)	1,425 (69.17)	531 (74.27)	229 (70.90)	
Anticoagulation, n (%)						<0.001
No	1,055 (21.06)	394 (20.61)	474 (23.01)	138 (19.30)	49 (15.17)	
Yes	3,955 (78.94)	1,518 (79.39)	1,586 (76.99)	577 (80.70)	274 (84.83)	
ACEI/ARB, n (%)						<0.001
No	2,006 (40.04)	829 (43.36)	806 (39.13)	246 (34.41)	125 (38.70)	
Yes	3,004 (59.96)	1,083 (56.64)	1,254 (60.87)	469 (65.59)	198 (61.30)	
Outcomes						
In-hospital all-cause mortality						<0.001
Alive	4,860 (97.01)	1,833 (95.87)	2,028 (98.45)	703 (98.32)	296 (91.64)	
Death	150 (2.99)	79 (4.13)	32 (1.55)	12 (1.68)	27 (8.36)	
10-year all-cause mortality						<0.001
Alive	4,375 (87.33)	1,629 (85.20)	1,879 (91.21)	634 (88.67)	233 (72.14)	
Death	635 (12.67)	283 (14.80)	181 (8.79)	81 (11.33)	90 (27.86)	

Continuous variables are presented as mean ± SD if normally distributed, and median (interquartile range) if not normally distributed. Categorical variables are presented as number of patients (%). P values were calculated using one-way ANOVA, Kruskal-Wallis test, or chi-squared test as appropriate. cTnT, cardiac troponin T; CK, creatine kinase; CK-MB, creatine kinase-MB; CRP, C-reactive protein; WBC, white blood cell; UN, urea nitrogen; LDL-C, low-density lipoprotein cholesterol; HDL-C, high-density lipoprotein cholesterol; ACEI, angiotensin-converting enzyme inhibitor; ARB, angiotensin II receptor blocker; NSTEMI, non-ST-segment elevation myocardial infarction; BMI, body mass index; TAMI, Tianjin Inpatient Acute Myocardial Infarction.

### 3.2 U-shaped association of BMI with all-cause mortality

**Figure 2:**
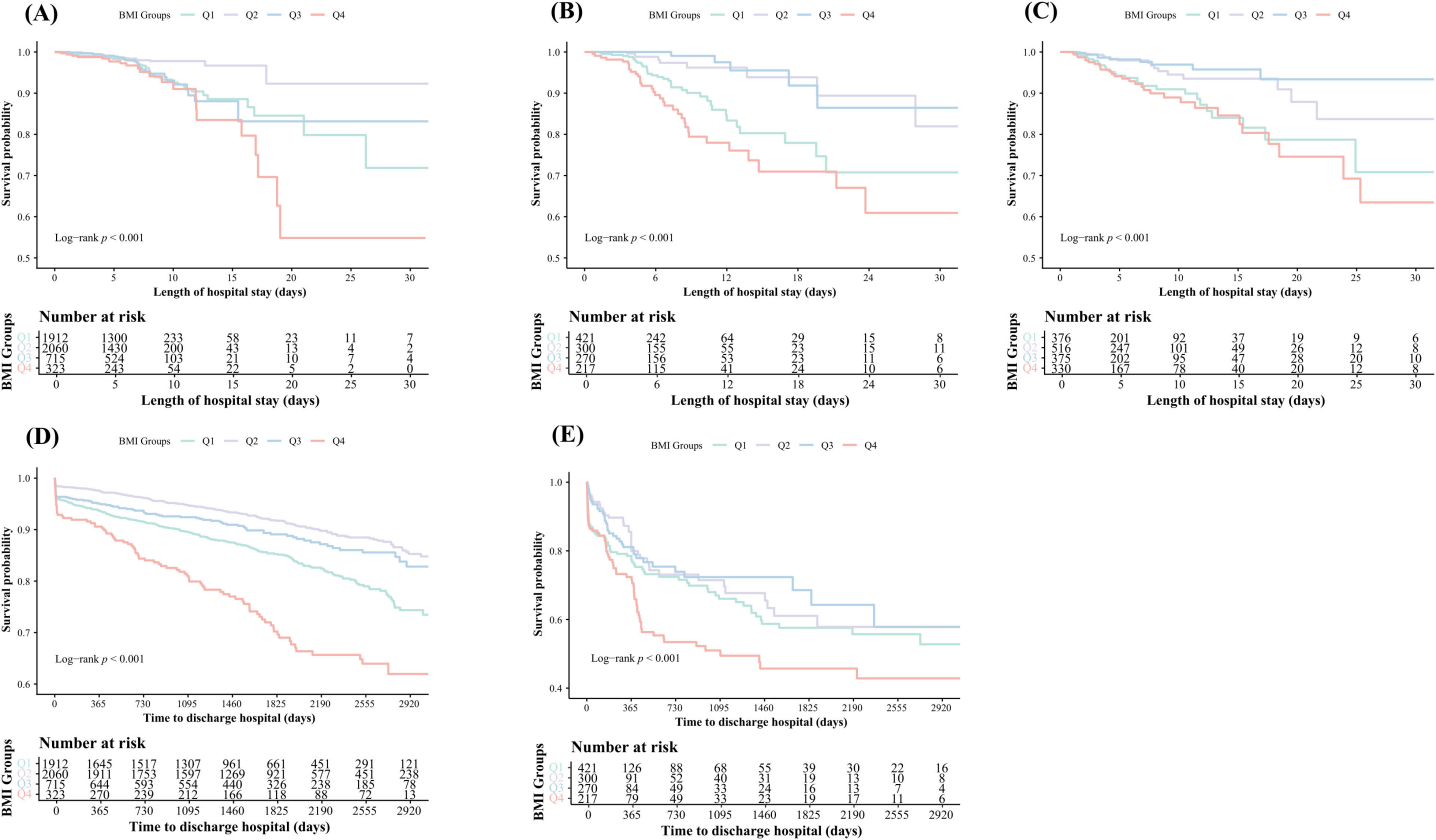
Kaplan-Meier curves for risk of all-cause mortality in NSTEMI patients grouped by BMI level. Q1: normal (BMI 18.5-24.9 kg/m^2^); Q2: overweight (BMI 25.0-29.9 kg/m^2^); Q3: obesity I (BMI 30.0-34.9 kg/m^2^); Q4: severe obesity (BMI ≥ 35.0 kg/m^2^). **(A)** BMI and in-hospital all-cause mortality in the TAMI cohort; **(B)** BMI and in-hospital all-cause mortality in the MIMIC-IV cohort; **(C)** BMI and in-hospital all-cause mortality in the eICU-CRD cohort; **(D)** BMI and 10-year all-cause mortality in the TAMI cohort; **(E)** BMI and 10-year all-cause mortality in the MIMIC-IV cohort. NSTEMI, non-ST-segment elevation myocardial infarction; BMI, body mass index; TAMI, Tianjin Inpatient Acute Myocardial Infarction; MIMIC-IV, Medical Information Mart for Intensive Care IV; eICU-CRD, eICU Collaborative Research Database.

The K-M survival curves revealed that for both in-hospital and 10-year all-cause mortality, the severe obesity group consistently demonstrated the poorest survival. In contrast, overweight and obesity I were associated with lower mortality risk compared with normal weight (**Figure 2**). Although the K-M curves showed some early crossing, formal testing using Schoenfeld residuals confirmed no violation of the proportional hazards assumption (global *p* > 0.05 for all models; **Supplementary Table 4**), indicating that the Cox regression results are reliable.

For in-hospital all-cause mortality, after full adjustment (Model 3), risk reductions in the overweight group were 50%, 56%, and 50% in the TAMI, MIMIC-IV, and eICU-CRD cohorts, respectively (hazard ratio (HR) 0.50; 95% CI: 0.33-0.76; *p* = 0.001; HR 0.44; 95% CI: 0.21-0.89; *p* = 0.022; HR 0.50; 95% CI: 0.27-0.93; *p* = 0.027). Corresponding reductions for obesity I were 55%, 75%, and 66% (HR 0.45; 95% CI: 0.24-0.83; *p* = 0.011; HR 0.25; 95% CI, 0.11-0.57; *p* < 0.001; HR 0.34; 95% CI: 0.16-0.72; *p* = 0.005). Conversely, severe obesity was associated with increased risks of 69%, 60%, and 80% (HR 1.69; 95% CI: 1.08-2.65; *p* = 0.022; HR 1.60; 95% CI: 1.02-2.52; *p* = 0.039; HR 1.80; 95% CI, 1.04-3.14; *p* < 0.001). Following BMI dichotomization, patients with severe obesity showed substantially elevated mortality risks (HR 2.37; 95% CI, 1.55-3.61; *p* < 0.001; HR 2.66; 95% CI: 1.78-3.98; *p* < 0.001; HR 3.01; 95% CI: 1.86-4.86; *p* < 0.001).

For 10-year all-cause mortality, risk reductions in the overweight group were 41%, and 40% in the TAMI and MIMIC-IV cohorts, respectively (HR 0.59; 95% CI: 0.48-0.71; *p* < 0.001; HR 0.60; 95% CI: 0.42-0.85; *p* = 0.004). Corresponding reductions for obesity I were 26% and 39% (HR 0.74; 95% CI: 0.58-0.95; *p* = 0.017; HR 0.61; 95% CI: 0.43-0.88; *p* = 0.008). Conversely, severe obesity was associated with increased risks of 68%, and 56% (HR 1.68; 95% CI: 1.31-2.15; *p* < 0.001; HR 1.56; 95% CI: 1.14-2.15; *p* = 0.006). Following BMI dichotomization, patients with severe obesity showed substantially elevated mortality risks (HR 2.18; 95% CI: 1.73-2.76; *p* < 0.001; HR 2.04; 95% CI: 1.53-2.73; *p* < 0.001). Detailed results are shown in **Table 2**.

**Table 2:** Multivariable-adjusted HR (95% CI) of BMI and all-cause mortality in the three cohorts.

BMI	Event/Total (%)	Model 1	p value	Model 2	p value	Model 3	p value
		HR (95% CI)		HR (95% CI)		HR (95% CI)	
TAMI cohort							
In-hospital all-cause mortality							
BMI (continuous)	150/5010 (2.99)	1.03 (1.01,1.06)	0.031	1.04 (1.01,1.07)	0.015	1.04 (1.01,1.07)	0.013
BMI (four categories)							
Normal (18.5-24.9)	79/1,912 (4.13)	Reference		Reference		Reference	
Overweight (25.0-29.9)	32/2,060 (1.55)	0.41 (0.27,0.61)	<0.001	0.49 (0.32,0.74)	<0.001	0.50 (0.33,0.76)	0.001
Obesity I (30.0-34.9)	12/715 (1.68)	0.39 (0.21,0.71)	0.002	0.46 (0.25,0.84)	0.012	0.45 (0.24,0.83)	0.011
Severe obesity (≥ 35.0)	27/323 (8.36)	1.62 (1.04,2.51)	0.032	1.69 (1.09,2.62)	0.019	1.69 (1.08,2.65)	0.022
BMI (two categories)							
Non-severe obesity (< 35.0)	125/4,687 (2.67)	Reference		Reference		Reference	
Severe obesity (≥ 35.0)	27/323 (8.36)	2.48 (1.63,3.78)	<0.001	2.34 (1.54,3.55)	<0.001	2.37 (1.55,3.61)	<0.001
10-year all-cause mortality							
BMI (continuous)	635/5,010 (12.67)	1.03 (1.01,1.05)	<0.001	1.04 (1.03,1.06)	<0.001	1.03 (1.01,1.05)	<0.001
BMI (four categories)							
Normal (18.5-24.9)	283/1,912 (14.80)	Reference		Reference		Reference	
Overweight (25.0-29.9)	181/2,060 (8.79)	0.52 (0.44,0.63)	<0.001	0.65 (0.54,0.78)	<0.001	0.59 (0.48,0.71)	<0.001
Obesity I (30.0-34.9)	81/715 (11.33)	0.67 (0.52,0.85)	0.001	0.86 (0.67,1.09)	0.219	0.74 (0.58,0.95)	0.017
Severe obesity (≥ 35.0)	90/323 (27.86)	1.66 (1.29,2.12)	<0.001	1.98 (1.55,2.54)	<0.001	1.68 (1.31,2.15)	<0.001
BMI (two categories)							
Non-severe obesity (< 35.0)	545/4,687 (11.63)	Reference		Reference		Reference	
Severe obesity (≥ 35.0)	90/323 (27.86)	2.29 (1.81,2.88)	<0.001	2.39 (1.89,3.02)	<0.001	2.18 (1.73,2.76)	<0.001
MIMIC-IV cohort							
In-hospital all-cause mortality							
BMI (continuous)	88/1,208 (7.28)	1.03 (1.01,1.06)	0.009	1.03 (1.01,1.06)	0.006	1.03 (1.01,1.06)	0.015
BMI (four categories)							
Normal (18.5-24.9)	38/421 (9.03)	Reference		Reference		Reference	
Overweight (25.0-29.9)	9/300 (3.00)	0.42 (0.21,0.86)	0.017	0.42 (0.21,0.86)	0.017	0.44 (0.21,0.89)	0.022
Obesity I (30.0-34.9)	6/270 (2.22)	0.26 (0.12,0.59)	0.001	0.25 (0.11,0.56)	<0.001	0.25 (0.11,0.57)	<0.001
Severe obesity (≥ 35.0)	35/217 (16.13)	1.58 (1.01,2.45)	0.045	1.65 (1.05,2.57)	0.029	1.60 (1.02,2.52)	0.039
BMI (two categories)							
Non-severe obesity (< 35.0)	53/991 (5.35)	Reference		Reference		Reference	
Severe obesity (≥ 35.0)	35/217 (16.13)	2.69 (1.82,3.98)	<0.001	2.84 (1.92,4.22)	<0.001	2.66 (1.78,3.98)	<0.001
10-year all-cause mortality							
BMI (continuous)	260/1,208 (21.52)	1.01 (0.99,1.03)	0.323	1.01 (0.99,1.03)	0.106	1.01 (0.99,1.03)	0.118
BMI (four categories)							
Normal (18.5-24.9)	99/421 (23.52)	Reference		Reference		Reference	
Overweight (25.0-29.9)	46/300 (15.33)	0.66 (0.47,0.94)	0.021	0.61 (0.43,0.87)	0.006	0.60 (0.42,0.85)	0.004
Obesity I (30.0-34.9)	42/270 (15.56)	0.67 (0.47,0.96)	0.029	0.62 (0.43,0.89)	0.011	0.61 (0.43,0.88)	0.008
Severe obesity (≥ 35.0)	73/217 (33.64)	1.36 (1.01,1.84)	0.046	1.47 (1.09,1.99)	0.013	1.56 (1.14,2.15)	0.006
BMI (two categories)							
Non-severe obesity (< 35.0)	187/991 (18.87)	Reference		Reference		Reference	
Severe obesity (≥ 35.0)	73/217 (33.64)	1.68 (1.28,2.20)	<0.001	1.89 (1.44,2.49)	<0.001	2.04 (1.53,2.73)	<0.001
eICU-CRD cohort							
In-hospital all-cause mortality							
BMI (continuous)	89/1,597 (5.57)	1.03 (1.01,1.06)	0.024	1.06 (1.03,1.08)	<0.001	1.05 (1.02,1.08)	<0.001
BMI (four categories)							
Normal (18.5-24.9)	32/376 (8.51)	Reference		Reference		Reference	
Overweight (25.0-29.9)	16/516 (3.10)	0.38 (0.21,0.70)	0.002	0.47 (0.26,0.87)	0.017	0.50 (0.27,0.93)	0.027
Obesity I (30.0-34.9)	9/375 (2.40)	0.25 (0.12,0.53)	<0.001	0.34 (0.16,0.72)	0.005	0.34 (0.16,0.72)	0.005
Severe obesity (≥ 35.0)	32/330 (9.70)	1.16 (0.71,1.90)	0.545	1.84 (1.08,3.15)	0.025	1.80 (1.04,3.14)	0.038
BMI (two categories)							
Non-severe obesity (< 35.0)	57/1,267 (4.50)	Reference		Reference		Reference	
Severe obesity (≥ 35.0)	32/330 (9.70)	2.24 (1.45,3.46)	<0.001	3.14 (1.99,4.96)	<0.001	3.01 (1.86,4.86)	<0.001

Model 1: unadjusted; Model 2: adjusted for age and sex; Model 3: further adjusted for hypertension, diabetes mellitus, hyperlipidemia, stroke, and atrial fibrillation. BMI, body mass index; HR, hazard ratio; CI, confidence interval; TAMI, Tianjin Inpatient Acute Myocardial Infarction; MIMIC-IV, Medical Information Mart for Intensive Care IV; eICU-CRD, eICU Collaborative Research Database.

We also examined the absolute event rates. In the TAMI cohort, the in-hospital all-cause mortality rate was lowest in the overweight group (1.55%) and highest in the severe obesity group (8.36%). For 10-year all-cause mortality, the absolute rates followed a similar pattern, ranging from 8.79% in the overweight group to 27.86% in the severe obesity group (**Table 2**). Consistent patterns were observed in the MIMIC-IV and eICU-CRD cohorts.

**Figure 3:**
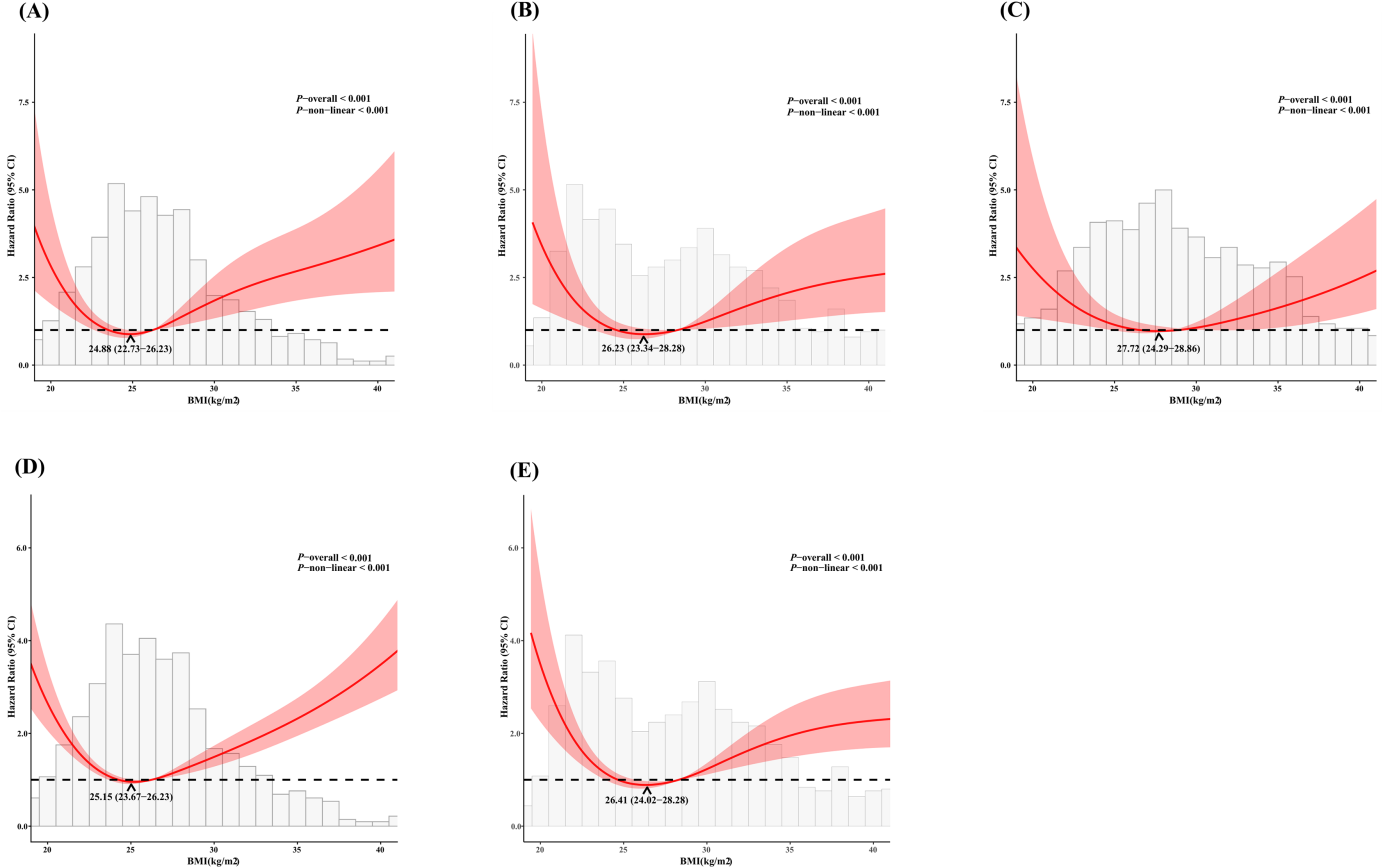
Restricted cubic spline plots for risk of all-cause mortality according to BMI on a continuous scale. Solid lines represent multivariable-adjusted hazard ratios, with shaded areas showing 95% confidence intervals (CI) derived from restricted cubic spline (RCS) regressions with four knots. Reference lines for no association are indicated by dashed black lines at a hazard ratio of 1.0. **(A)** BMI and in-hospital all-cause mortality in the TAMI cohort; **(B)** BMI and in-hospital all-cause mortality in the MIMIC-IV cohort; **(C)** BMI and in-hospital all-cause mortality in the eICU-CRD cohort; **(D)** BMI and 10-year all-cause mortality in the TAMI cohort; **(E)** BMI and 10-year all-cause mortality in the MIMIC-IV cohort. The nadir of mortality risk was observed at the following BMI values (95% CI): **(A)** 24.88 (22.73-26.23) kg/m^2^; **(B)** 26.23 (23.34-28.28) kg/m^2^; **(C)** 27.72 (24.29-28.86) kg/m^2^; **(D)** 25.15 (23.67-26.23) kg/m^2^; **(E)** 26.41 (24.02-28.28) kg/m^2^. Mortality risk across these BMI ranges did not differ significantly from the respective nadir. Models were adjusted for all variables in Model 3. BMI, body mass index; CI, confidence interval; RCS, restricted cubic spline; TAMI, Tianjin Inpatient Acute Myocardial Infarction; MIMIC-IV, Medical Information Mart for Intensive Care IV; eICU-CRD, eICU Collaborative Research Database.

RCS analysis revealed a significant non-linear, U-shaped relationship between BMI and all-cause mortality (all *p* for non-linearity <0.001). The nadir of risk for in-hospital all-cause mortality was at 24.88, 26.23, and 27.72 kg/m^2^, while for 10-year all-cause mortality, the nadir was at 25.15 and 26.41 kg/m^2^, indicating that the point of minimal mortality risk consistently fell within the overweight range (**Figure 3**).

### 3.3 Joint association of BMI and CRP with all-cause mortality

The K-M survival curves showed that the high BMI/high CRP group consistently demonstrated the poorest survival across all cohorts (**Supplementary Figure 4**).

For in-hospital all-cause mortality, compared with the reference group (low BMI/low CRP), the low BMI/high CRP group was associated with 1.51-fold (TAMI),1.89-fold (MIMIC-IV) and 2.20-fold (eICU-CRD) increased risks (HR 1.51; 95% CI: 1.03-2.20; *p* = 0.034; HR 1.89; 95% CI: 1.04-3.46; *p* = 0.041; HR 2.20; 95% CI: 1.21-4.01; *p* = 0.011). The high BMI/low CRP group showed 2.26-fold, 3.00-fold and 3.06-fold increases (HR 2.26; 95% CI: 1.15-4.42; *p* = 0.018; HR 3.00; 95% CI: 1.46–6.18; *p* = 0.003; HR 3.06; 95% CI: 1.25-7.47; *p* = 0.014). The highest risk was observed in the high BMI/high CRP group, with 3.86-fold, 3.86-fold and 3.62-fold increases (HR 3.86; 95% CI: 2.11-7.07; *p* < 0.001; HR 3.86; 95% CI: 2.07-7.20; *p* < 0.001; HR 3.62; 95% CI: 1.74-7.56; *p* < 0.001).

For 10-year all-cause mortality, the low BMI/high CRP group showed 1.41-fold and 1.90-fold increases (HR 1.41; 95% CI: 1.18-1.68; *p* < 0.001; HR 1.90; 95% CI: 1.36-2.65; *p* < 0.001). The high BMI/low CRP group showed 2.37-fold and 2.50-fold increases (HR 2.37; 95% CI: 1.67-3.38; *p* < 0.001; HR 2.50; 95% CI: 1.43-4.36; *p* < 0.001). The high BMI/high CRP group showed 3.06-fold and 3.45-fold increases (HR 3.06; 95% CI: 2.20-4.24; *p* < 0.001; HR 3.45; 95% CI: 2.30-5.17; *p* < 0.001). Details results are shown in **Table 3**.

**Table 3:** Multivariable-adjusted HR (95% CI) of BMI combined with CRP and all-cause mortality in the three cohorts.

Characteristics	Event/Total (%)	Model 1	p value	Model 2	p value	Model 3	p value
		HR (95% CI)		HR (95% CI)		HR (95% CI)	
TAMI cohort							
In-hospital all-cause mortality	150/5,010 (2.99)						
BMI <35.0 kg/m2 and CRP <2 mg/L	40/2,073 (1.93)	Reference		Reference		Reference	
BMI <35.0 kg/m2 and CRP ≥2 mg/L	84/2,614 (3.21)	1.57 (1.08,2.29)	0.019	1.55 (1.06,2.26)	0.023	1.51 (1.03,2.20)	0.034
BMI ≥35.0 kg/m2 and CRP <2 mg/L	11/179 (6.15)	2.49 (1.28,4.86)	0.008	2.25 (1.15,4.39)	0.018	2.26 (1.15,4.42)	0.018
BMI ≥35.0 kg/m2 and CRP ≥2 mg/L	15/144 (10.42)	4.03 (2.22,7.32)	<0.001	3.90 (2.14,7.09)	<0.001	3.86 (2.11,7.07)	<0.001
10-year all-cause mortality	635/5,010 (12.67)						
BMI <35.0 kg/m2 and CRP <2 mg/L	190/2,073 (9.17)	Reference		Reference		Reference	
BMI <35.0 kg/m2 and CRP ≥2 mg/L	355/2,614 (13.58)	1.47 (1.23,1.76)	<0.001	1.41 (1.18,1.68)	<0.001	1.41 (1.18,1.68)	<0.001
BMI ≥35.0 kg/m2 and CRP <2 mg/L	37/179 (20.67)	2.40 (1.69,3.42)	<0.001	2.63 (1.85,3.74)	<0.001	2.37 (1.67,3.38)	<0.001
BMI ≥35.0 kg/m2 and CRP ≥2 mg/L	53/144 (36.81)	3.48 (2.52,4.82)	<0.001	3.30 (2.38,4.59)	<0.001	3.06 (2.20,4.24)	<0.001
MIMIC-IV cohort							
In-hospital all-cause mortality	88/1,208 (7.28)						
BMI <35.0 kg/m2 and CRP <2 mg/L	11/414 (2.66)	Reference		Reference		Reference	
BMI <35.0 kg/m2 and CRP ≥2 mg/L	42/577 (7.28)	1.92 (1.06,3.50)	0.031	1.96 (1.07,3.57)	0.031	1.89 (1.04,3.46)	0.041
BMI ≥35.0 kg/m2 and CRP <2 mg/L	10/75 (13.33)	3.13 (1.54,6.35)	0.002	3.23 (1.59,6.58)	0.001	3.00 (1.46,6.18)	0.003
BMI ≥35.0 kg/m2 and CRP ≥2 mg/L	25/142 (17.61)	3.81 (2.07,7.04)	<0.001	4.06 (2.19,7.53)	<0.001	3.86 (2.07,7.20)	<0.001
10-year all-cause mortality	260/1,208 (21.52)						
BMI <35.0 kg/m2 and CRP <2 mg/L	46/414 (11.11)	Reference		Reference		Reference	
BMI <35.0 kg/m2 and CRP ≥2 mg/L	141/577 (24.44)	2.06 (1.48,2.88)	<0.001	1.93 (1.38,2.70)	<0.001	1.90 (1.36,2.65)	<0.001
BMI ≥35.0 kg/m2 and CRP <2 mg/L	22/75 (29.33)	2.17 (1.26,3.75)	0.005	2.30 (1.33,3.98)	0.003	2.50 (1.43,4.36)	0.001
BMI ≥35.0 kg/m2 and CRP ≥2 mg/L	51/142 (35.92)	3.01 (2.03,4.46)	<0.001	3.27 (2.21,4.85)	<0.001	3.45 (2.30,5.17)	<0.001
eICU-CRD cohort							
In-hospital all-cause mortality	89/1,597 (5.57)						
BMI <35.0 kg/m2 and CRP <2 mg/L	14/529 (2.65)	Reference		Reference		Reference	
BMI <35.0 kg/m2 and CRP ≥2 mg/L	50/745 (6.71)	2.52 (1.39,4.56)	0.002	2.36 (1.30,4.28)	0.005	2.20 (1.21,4.01)	0.011
BMI ≥35.0 kg/m2 and CRP <2 mg/L	8/134 (5.97)	2.32 (0.97,5.55)	0.058	3.24 (1.34,7.83)	0.009	3.06 (1.25,7.47)	0.014
BMI ≥35.0 kg/m2 and CRP ≥2 mg/L	17/189 (8.99)	3.44 (1.69,6.99)	<0.001	4.09 (2.00,8.37)	<0.001	3.62 (1.74,7.56)	<0.001

BMI categories were defined as < 35.0 kg/m^2^ (non-obesity II/III) and ≥ 35.0 kg/m^2^ (obesity II/III). CRP categories were defined as < 2 mg/L *vs*. ≥ 2 mg/L. Model 1: unadjusted; Model 2: adjusted for age and sex; Model 3: further adjusted for hypertension, diabetes mellitus, hyperlipidemia, stroke, and atrial fibrillation. BMI, body mass index; CRP, C-reactive protein; HR, hazard ratio; CI, confidence interval; TAMI, Tianjin Inpatient Acute Myocardial Infarction; MIMIC-IV, Medical Information Mart for Intensive Care IV; eICU-CRD, eICU Collaborative Research Database.

The highest absolute risk was consistently observed in the high BMI/high CRP group. In the TAMI cohort, the in-hospital all-cause mortality rate was 10.42% for this group, compared to only 1.93% for the low BMI/low CRP reference group. Similarly, for 10-year all-cause mortality, the high BMI/high CRP group had a rate of 36.81%, versus 9.17% for the reference group. Consistent patterns were observed in the MIMIC-IV and eICU-CRD cohorts.

No significant interaction was observed between cohort and severe obesity (*p* for interaction = 0.978) or between cohort and elevated CRP (*p* for interaction = 0.292) for in-hospital all-cause mortality. Similarly, for 10-year all-cause mortality, no significant interaction was found between cohort and either exposure (all *p* for interaction > 0.05). These findings indicate that the associations between severe obesity, elevated CRP, and all-cause mortality are consistent across the three cohorts (**Supplementary Table 9**).

To formally evaluate whether the combined effect exceeded additive or multiplicative expectations, we performed formal interaction tests. The product term was not statistically significant in any cohort (all *p* > 0.05). Additive interaction metrics (RERI, AP, and SI) showed positive point estimates, but their 95% CIs crossed the null value (0 for RERI and AP, 1 for SI). Thus, neither multiplicative nor additive interaction reached statistical significance (**Supplementary Table 10**).

Nevertheless, the stratified analysis consistently demonstrated that patients with both severe obesity and elevated CRP (the high BMI/high CRP group) had the highest mortality risk across all cohorts and outcomes, identifying a distinct high-risk clinical subgroup.

### 3.4 Subgroup analysis

Prespecified subgroup analyses were performed by age (<80/≥80 years), sex (male/female), HBP (yes/no), and DM (yes/no). The increased mortality risk associated with severe obesity was more pronounced in males, those aged <80 years, and those with HBP or DM, for both in-hospital and 10-year all-cause mortality. Consistently, the elevated risk associated with the combined high BMI/high CRP was most evident in these same subgroups.

Notably, the combined presence of severe obesity, elevated inflammation, and DM was associated with the highest all-cause mortality risk, with adjusted HRs reaching 4.98, 4.74 and 4.78 (95% CI: 2.51-9.86; *p* < 0.001; 95% CI: 1.72-8.22, *p* = 0.008; 95% CI: 1.62-7.06, *p* = 0.005) for in-hospital mortality, and 3.61 and 3.95 (95% CI: 2.44-5.32; *p* < 0.001; 95% CI: 1.69-6.87; *p* = 0.001) for 10-year all-cause mortality. These results identified a unique subgroup with extreme mortality risk. Detailed results are shown in **Supplementary Figures 5**-**8**.

We performed interaction tests to evaluate effect modification by DM. No significant interactions were observed (all *p* > 0.05), indicating that the joint effect of severe obesity and elevated CRP on mortality was consistent across DM and non-DM patients (**Supplementary Table 11**).

### 3.5 Incremental value of CRP

Adding CRP to a comprehensive clinical model significantly improved risk prediction. In the TAMI cohort, the addition of CRP improved the AUC for in-hospital all-cause mortality from 0.764 to 0.768 (*p* = 0.021), with an NRI of 24.24% (95% CI: 9.52%-38.97%, *p* = 0.001) and IDI of 0.61% (95% CI: 0.03%-1.18%, *p* = 0.037). For 10-year all-cause mortality, the AUC improved from 0.796 to 0.801 (*p* < 0.001), with an NRI of 21.08% (95% CI: 13.06%-29.10%, *p* < 0.001) and IDI of 0.29% (95% CI: 0.08%-0.49%, *p* = 0.006). In the MIMIC-IV and eICU-CRD cohorts, similar significant improvements were observed. Details are shown in **Supplementary Table 12**.

Calibration curves for both models closely followed the diagonal reference line across all cohorts and outcomes (**Supplementary Figure 9**). The calibration slope ranged from 0.92 to 0.98, and Emax was consistently < 0.05. The curves were nearly identical between models, indicating that adding CRP did not compromise calibration. These findings, together with the improvements in AUC, NRI, and IDI, confirm that CRP adds meaningful incremental prognostic value.

### 3.6 Mediation analysis

CRP significantly mediated the association between severe obesity and all-cause mortality across all three cohorts. For in-hospital all-cause mortality, the proportion mediated ranged from 13.7% to 16.9%. For 10-year all-cause mortality, the proportion mediated was 8.5% and 9.6% in the two cohorts with available long-term follow-up. The mediating effect was consistently stronger for short-term mortality, reflecting the acute inflammatory burden captured by CRP at admission (**Supplementary Figure 10**).

### 3.7 Sensitivity analysis

Eight sensitivity analyses, including competing risk regression, exclusion of early deaths, additional adjustment for continuous BMI, adjustment for additional covariates, complete-case analysis for missing CRP, application of Chinese BMI criteria, and meta-analysis, all yielded results consistent with the primary analysis (**Supplementary Tables 13**–**19**; **Supplementary Figure 11**). E-value analysis confirmed robustness to unmeasured confounding, with E-values ranging from 1.77 to 4.56 across different BMI categories and outcomes (**Supplementary Table 16**). All sensitivity analyses supported the robustness of the primary findings.

## 4 Discussion

A major strength of this study is its three-cohort validation design: an original discovery cohort (TAMI) with external validation in two independent cohorts (MIMIC-IV, eICU-CRD), yielding consistent results and meeting the requirement for independent clinical validation. In this multinational study encompassing three international cohorts, we investigated the association between BMI and all-cause mortality, both in-hospital and at 10 years, among critically ill NSTEMI patients admitted to the CCU, with a particular focus on the incremental role of the inflammatory biomarker CRP. Our principal findings reveal a consistent U-shaped relationship between BMI and mortality across cohorts, with the nadir of risk residing within the overweight range. Overweight and obesity I were associated with lower mortality risk compared with normal weight, whereas severe obesity was associated with a substantially increased mortality risk. Critically, the combination of severe obesity and elevated CRP identified a distinct clinical profile at exceptionally high risk, suggesting that chronic metabolic derangements and acute inflammatory pathways may jointly contribute to poor prognosis. Subgroup analyses further highlighted that patients with the combined burden of severe obesity, elevated inflammation, and DM constituted an extreme-risk group. The robustness of our findings was reinforced by validation in two ethnically diverse external cohorts and a series of sensitivity analyses (Graphical Abstract).

Despite differences in ethnicity, ICU admission criteria, treatment practices, follow-up procedures, and BMI distribution across the three cohorts, formal interaction tests revealed no significant heterogeneity in the associations between severe obesity or elevated CRP and all-cause mortality. The consistency of our findings across three independent cohorts with diverse healthcare settings and populations supports the generalizability of our conclusions.

The association between severe obesity and elevated mortality in critically ill NSTEMI patients is underpinned by a complex interplay of chronic adipose tissue pathology and acute metabolic-inflammatory stress. Under conditions of severe obesity, adipose tissue expansion occurs mainly via hypertrophy (enlargement of existing fat cells) rather than hyperplasia (new cell formation), causing significant changes in adipocyte quantity and quality (31). This abnormal expansion outpaces angiogenesis, resulting in inadequate local oxygen supply and abnormal lipid metabolism, which ultimately induces adipocyte death (32). The resulting pathological microenvironment triggers massive infiltration of immune cells, particularly pro-inflammatory macrophages, which become a major source of pro-inflammatory cytokines (33). This drives metabolic inflammation within adipose tissue and establishes a state of chronic low-grade systemic inflammation. Such a baseline inflammatory milieu promotes endothelial dysfunction, insulin resistance, and a pro-thrombotic state, fundamental processes in atherosclerosis progression and plaque vulnerability (34). Visceral adipose tissue (VAT) accumulation represents a hallmark of ectopic fat deposition and metabolic dysregulation. During acute NSTEMI, VAT lipolysis releases large quantities of free fatty acids and inflammatory mediators, which not only exacerbate systemic insulin resistance but also directly impair myocardial metabolic function (35). The ischemic and hypoxic myocardium is forced to rely on these inefficient fatty acid substrates. Coupled with increased oxygen consumption during NSTEMI, this leads to mitochondrial dysfunction and accumulation of toxic lipid intermediates such as ceramides, resulting in lip toxicity and ultimately accelerated cardiomyocyte apoptosis (36).

Our findings demonstrate that NSTEMI patients with severe obesity and concomitant elevated inflammation (CRP ≥ 2 mg/L) face a significantly amplified risk of all-cause mortality, identifying a distinct high-risk clinical subgroup. The acute NSTEMI event triggers a systemic inflammatory response, which in the setting of severe obesity is superimposed on a pre-existing, obesity-driven chronic low-grade inflammation emanating from dysfunctional VAT (37). This combined burden triggers a vicious cycle of exacerbated lipolysis, inflammatory mediator release, and metabolic stress, which likely aggravates endothelial injury, plaque instability, and microvascular dysfunction, culminating in the observed increase in mortality (38, 39). Thus, the joint presence of these two factors defines an exceptionally vulnerable population that warrants targeted monitoring and intervention.

With the widespread adoption of emergency PCI for STEMI, NSTEMI has emerged as a more common and clinically intricate cardiovascular emergency compared with STEMI. Extreme BMI values are established risk factors for numerous conditions. Severe obesity, in particular, is a key driver of metabolic disorders and CVD progression (40). The association between BMI and prognosis after AMI has been debated, with some studies describing an ‘obesity paradox’ wherein overweight or mildly obese patients show reduced mortality. However, this apparent protective effect appears limited to a specific BMI range, and evidence focusing specifically on critically ill NSTEMI patients requiring CCU admission remains scarce (41). Our multinational study, targeting this high-risk cohort, clarifies this association by demonstrating a consistent U-shaped relationship between BMI and both in-hospital and 10-year all-cause mortality, with the nadir of risk in the overweight range and a sharply increased risk in severe obesity.

Our findings indicate that mortality risk begins to rise with obesity I and becomes significantly elevated with severe obesity. In this sense, our results support the concept that severe obesity is a modifiable risk factor not only for primary and secondary prevention, but also for tertiary prevention of cardiovascular events.

We acknowledge that BMI is an imperfect measure of adiposity, as it does not distinguish between fat mass and lean mass, visceral versus subcutaneous fat, or sarcopenic obesity. In the ICU/CCU setting, direct measurements of waist circumference, waist-to-hip ratio, or body composition are not routinely collected in large-scale retrospective databases. Despite this, the consistent U-shaped association between BMI and mortality observed across three independent cohorts, along with the robust E-value analysis supports the validity of our findings.

Smoking status and physical activity level were not included in the adjustment models due to incomplete or unreliable recording in the retrospective ICU databases. Both factors are well-established confounders in studies of obesity and cardiovascular outcomes. In critically ill NSTEMI patients, severe obesity is negatively associated with smoking (fewer current smokers among those with BMI ≥ 35 kg/m²), whereas smoking is positively associated with mortality (42). This bidirectional bias would lead to an underestimation of the true mortality risk of severe obesity, biasing the hazard ratio toward the null. Conversely, physical inactivity is positively associated with both severe obesity and mortality; its omission would tend to overestimate the obesity-related risk (43). However, part of the effect of physical inactivity is already captured by the adjusted mediators (e.g., HBP, DM, and HL). The consistent U-shaped association across three independent cohorts and the robustness of the sensitivity analyses (E-value showed that an unmeasured confounder would need a risk ratio of ≥ 2.24 to explain the protective effect of overweight) suggest that residual confounding from smoking and physical inactivity is unlikely to fully account for our findings.

Although we observed a significant mediating role of CRP, we acknowledge that elevated CRP may reflect greater disease severity rather than a specific causal pathway. First, acute myocardial injury burden can independently elevate CRP and worsen prognosis. Second, concurrent infections or systemic illness can independently elevate CRP and increase mortality. We adjusted for cTnT and WBC, and the results remained robust. Third, heart failure severity can trigger an acute-phase response. However, the lack of consistent data on left ventricular ejection fraction, NT-proBNP, and SOFA scores across the three cohorts limited our ability to perform unified adjustments. Future studies integrating serial inflammatory biomarkers, imaging-based myocardial injury assessment, and dynamic severity scores are needed to further elucidate the causal pathways.

The observed lower mortality risk associated with overweight and obesity I (the ‘obesity paradox’) warrants careful interpretation. Several non-causal explanations may contribute. First, selection bias may play a role. Although we excluded patients with BMI < 18.5 kg/m² to avoid confounding by cachexia, the remaining normal-weight group may still contain individuals who experienced disease-related weight loss from a previously higher BMI, artificially elevating its mortality rate (44). Second, reverse causation is a concern. Chronic subclinical diseases can cause unintentional weight loss before NSTEMI, reclassifying high-risk patients into the normal-weight category (45). Our sensitivity analysis excluding early deaths (**Supplementary Table 12**) yielded consistent results, suggesting that reverse causation is not the primary driver. Third, residual confounding from unmeasured variables may also contribute. These include illness severity scores (e.g., SOFA/APACHE), frailty, cachexia, nutritional status, smoking, malignancy, left ventricular ejection fraction, socioeconomic factors and weight loss preceding admission. Frailty and cachexia are associated with both lower BMI and higher mortality, inflating the risk in the normal-weight group. The higher mortality observed at the lower end of the BMI spectrum likely reflects underlying illness rather than a causal effect of leanness itself (46). In contrast, the increased risk at high BMI is more likely attributable to direct pathophysiological consequences of excess adiposity. Although E-value analyses (**Supplementary Table 14**) suggested robustness to unmeasured confounding, residual confounding cannot be completely ruled out. Therefore, the protective association for overweight and obesity I should be viewed as a risk-stratification phenomenon rather than a causal benefit (47).

We acknowledge that the 10-year all-cause mortality outcome is influenced by post-discharge factors not captured in our data, including changes in BMI over time, secondary prevention adherence, subsequent cardiovascular events, and evolving medical therapies over the 10-year follow-up period. Although these factors may confound the observed associations, the consistency of our findings across three independent cohorts and the robust E-value analysis suggest that unmeasured post-discharge factors are unlikely to fully explain our results. Future studies with longitudinal data on weight trajectories and recurrent events are needed to better understand the long-term impact of obesity and inflammation in this population.

Our subgroup analyses indicated that the mortality risk associated with the combined BMI-CRP was most pronounced in males, those under 80 years of age, and those with HBP or DM. Among these, the coexistence of severe obesity, elevated inflammation, and DM delineated a distinct extreme-risk cluster. DM and severe obesity exacerbate insulin resistance, elevate chronic low-grade inflammation, and impair endothelial function and energy metabolism. During acute NSTEMI, lipolysis of visceral adipose tissue floods the circulation with free fatty acids and inflammatory mediators, which, against a backdrop of pre-existing insulin resistance, further compromise myocardial metabolism and amplify the inflammatory response (48). A Taiwanese observational study of 1,193 patients with DM and acute coronary syndrome reported a U-shaped relationship between BMI and mortality, with the nadir at 27–30 kg/m^2^ (49). Another prospective observational study of 19,478 patients with DM found a U-shaped association between BMI and all-cause mortality, with severe obesity conferring a 2.21-fold increased mortality risk (50). In other subgroups, risk modulation appears to differ: in the elderly, age-related sarcopenia may convert a higher BMI into a marker of physiological reserve, thereby attenuating obesity-related risk (51). Males are likely more vulnerable due to greater visceral adipose tissue deposition and a more robust pro-inflammatory response, whereas females may be relatively protected by hormonal influences, although disparities in care patterns may offset the lower mortality risk associated with overweight or obesity I observed in females (52). HBP compounds mortality risk through synergistic sympathetic overactivation and renin-angiotensin-aldosterone system stimulation, exacerbating hemodynamic stress and inflammatory activation during acute ischemia (53).

Our findings advocate for a paradigm shift towards a comprehensive, lifelong ‘metabolic-inflammatory’ dual-pathway management strategy for high-risk NSTEMI survivors. This integrated approach should emphasize sustained weight management (focus on reducing VAT), optimization of glycemic control and metabolic benefits, and the implementation of long-term anti-inflammatory strategies. Concurrently, this patient population represents a critical cohort for evaluating the efficacy of novel targeted anti-inflammatory drugs and intensified metabolic regimens in the acute cardiovascular setting. The ultimate goal is to transform their identified pathophysiological vulnerability into a target for therapeutic improvement (54).

Several limitations of this study should be acknowledged. First, BMI was measured only at CCU admission and not monitored during follow-up, during which significant weight fluctuations may have occurred. Indeed, weight variability is common and has been closely linked to increased mortality in AMI patients (55). Second, body composition measures, such as fat distribution and waist circumference, were unavailable, which could provide additional insight into the obesity-mortality relationship. Third, the study period spanned 10 years, during which advances in NSTEMI management may have influenced the observed associations. Fourth, this study focused only on all-cause mortality and did not include major adverse cardiovascular events. Fifth, smoking, physical activity, socioeconomic factors and BMI change were not adjusted for due to incomplete data, but sensitivity analyses (including E-values) suggested that our findings are robust to unmeasured confounding. Sixth, the lower mortality risk associated with overweight and obesity I may be influenced by selection bias and reverse causation, although our sensitivity analysis excluding early deaths yielded consistent results. Seventh, CRP data were incomplete; a complete-case sensitivity analysis yielded consistent results, confirming robustness. Finally, residual confounding from other unmeasured variables cannot be completely ruled out.

## 5 Conclusion

BMI exhibits a U-shaped association with mortality in critically ill NSTEMI patients, with severe obesity representing an independent risk factor. The combination of severe obesity and elevated inflammation identifies a distinct, high-risk clinical profile. In clinical practice, integrated “metabolic-inflammatory” management should be prioritized. Targeted strategies combining long-term weight control with anti-inflammatory therapy warrant further exploration to improve short- and long-term prognosis in this vulnerable population.

## Data Availability

The original contributions presented in the study are included in the article/**Supplementary Material**. Further inquiries can be directed to the corresponding author.

## Glossary

BMI: body mass index
CVD: cardiovascular diseases
AMI: acute myocardial infarction
ICU: intensive care unit
HF: heart failure
AKI: acute kidney injury
DM: diabetes mellitus
STEMI: ST-segment elevation myocardial infarction
NSTEMI: non-ST-segment elevation myocardial infarction
CCU: coronary care unit
CRP: C-reactive protein
TAMI: Tianjin Inpatient Acute Myocardial Infarction
MIMC-IV: Medical Information Mart for Intensive Care IV
eICU-CRD: eICU Collaborative Research Database
CITI: collaborative institutional training initiative
HBP: hypertension
AF: atrial fibrillation
CK: creatine kinase
CK-MB: creatine kinase-MB
WBC: white blood cell
LDL-C: low-density lipoprotein cholesterol
HDL-C: high-density lipoprotein cholesterol
IABP: intra-aortic balloon pump
PCI: percutaneous coronary intervention
CABG: coronary-artery-bypass-grafting
ACEI: angiotensin-converting enzyme inhibitors
ARB: angiotensin receptor blockers
HIS: hospital information system
SQL: structured query language
WHO: World Health Organization
K-M: Kaplan-Meier
RCS: restricted cubic spline
VIF: Variance inflation factor
AUC: area under the curve
NRI: net reclassification improvement
IDI: integrated discrimination improvement
HRs: hazard ratios
VAT: Visceral adipose tissue.
