# Supplementary material for: Joint effects of severe obesity and inflammation on mortality in critically ill non−ST−segment elevation myocardial infarction patients: a cohort study with external validation
Source: Front Endocrinol (Lausanne). 2026 Jun 24;17:1850802. doi: 10.3389/fendo.2026.1850802 (PMC13341532; doi:10.3389/fendo.2026.1850802)
Supplement: Supplementary file 1 [file DataSheet1.pdf]

Supplementary Material

Supplementary Figure 1. RCS curves for CRP and all-cause mortality in the TAMI cohort.
Supplementary Figure 2. Determination of follow-up time for in-hospital and 10-year all-cause mortality.
Supplementary Figure 3. Directed acyclic graph.
Supplementary Figure 4. Kaplan-Meier curves for risk of all-cause mortality in NSTEMI patients grouped by BMI combined with CRP level.
Supplementary Figure 5. Subgroup analysis of BMI and in-hospital all-cause mortality in the three cohorts.
Supplementary Figure 6. Subgroup analysis of BMI and 10-year all-cause mortality in the two cohorts.
Supplementary Figure 7. Subgroup forest plots of BMI combined with CRP and in-hospital all-cause mortality in the three cohorts.
Supplementary Figure 8. Subgroup forest plots of BMI combined with CRP and 10-year all-cause mortality in the two cohorts.
Supplementary Figure 9. Calibration curves comparing the base model and the incremental model with CRP for predicting all-cause mortality.
Supplementary Figure 10. Mediation analyses of CRP in the association between severe obesity and all-cause mortality.
Supplementary Figure 11. Meta-analysis of BMI categories and all-cause mortality across the three cohorts.
Supplementary Table 1. Detailed information of three cohorts.
Supplementary Table 2. NSTEMI diagnostic criteria in three cohorts.
Supplementary Table 3. BMI groups in three cohorts.
Supplementary Table 4. Schoenfeld residual tests for proportional hazards assumption.
Supplementary Table 5. The variance inflation factor of covariates in the three cohorts.
Supplementary Table 6. Missing values of variables.
Supplementary Table 7. Baseline characteristics of all NSTEMI patients with different BMI levels in the MIMIC-IV cohort.
Supplementary Table 8. Baseline characteristics of all NSTEMI patients with different BMI levels in the eICU-CRD cohort.
Supplementary Table 9. Interaction tests between cohort and exposure for in-hospital and 10-year all-cause mortality.
Supplementary Table 10. Formal interaction tests between severe obesity and elevated CRP.
Supplementary Table 11. Interaction tests for severe obesity, elevated CRP, and diabetes for in-hospital and 10-year all-cause mortality.
Supplementary Table 12. Evaluation of predictive models for all-cause mortality.
Supplementary Table 13. Fine-Gray competing risk regression for BMI and 10-year all-cause mortality treating in-hospital death as a competing event.
Supplementary Table 14. Multivariate-adjusted HR (95% CI) of BMI and 10-year all-cause mortality after excluding patients who died within one year.
Supplementary Table 15. Multivariate-adjusted HR (95% CI) of BMI categories for all-cause mortality after additional adjustment for BMI as a continuous variable.

Supplementary Table 16. E-values for unmeasured confounding in the association between BMI categories and all-cause mortality.
Supplementary Table 17. Sensitivity analysis using Chinese obesity classification criteria for BMI and all-cause mortality in the TAMI cohort.
Supplementary Table 18. Multivariate-adjusted HR (95% CI) of BMI combined with CRP and all-cause mortality after excluding patients with missing CRP.
Supplementary Table 19. Multivariable-adjusted HR (95% CI) of BMI and all-cause mortality in the three cohorts (additional adjustment for PCI, CABG, cTnT and albumin).

Supplementary Figure 1. RCS curves for CRP and all-cause mortality in the TAMI cohort.

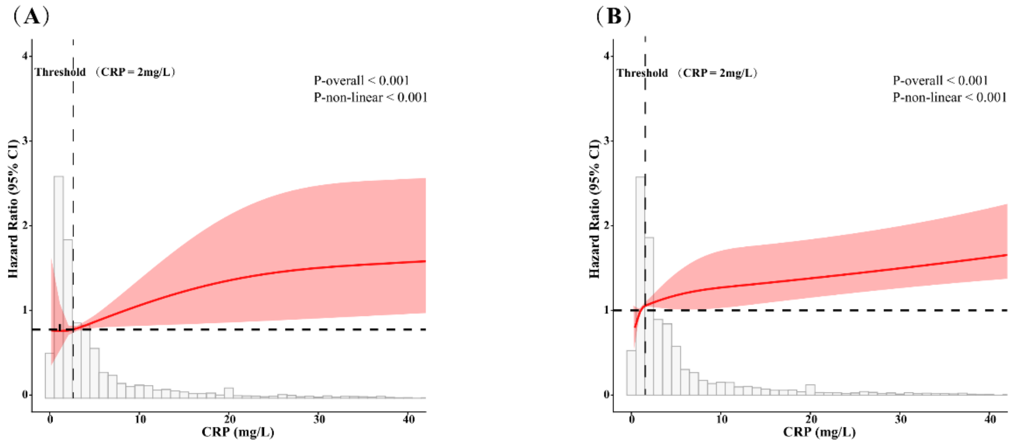

Notes: Solid lines represent multivariable-adjusted hazard ratios, with shaded areas showing 95% confidence intervals (CI) derived from restricted cubic spline (RCS) regressions with four knots. Reference lines for no association are indicated by dashed black lines at a hazard ratio of 1.0. (A): CRP and in-hospital all-cause mortality in the TAMI cohort; (B): CRP and 10-year all-cause mortality in the TAMI cohort. The 95% CI of the hazard ratio (HR) exceeds 1 at CRP > 2.09 mg/L in both panels, supporting the clinical relevance of the 2 mg/L threshold used in the primary analysis. The vertical dashed line at 2 mg/L indicates the prespecified threshold based on prior landmark trials (JUPITER, CANTOS). Models were adjusted for all variables in Model 3. CRP, C-reactive protein; HR, hazard ratio; CI, confidence interval; RCS, restricted cubic spline; TAMI, Tianjin Inpatient Acute Myocardial Infarction.

Supplementary Figure 2. Determination of follow-up time for in-hospital and 10-year all-cause mortality.

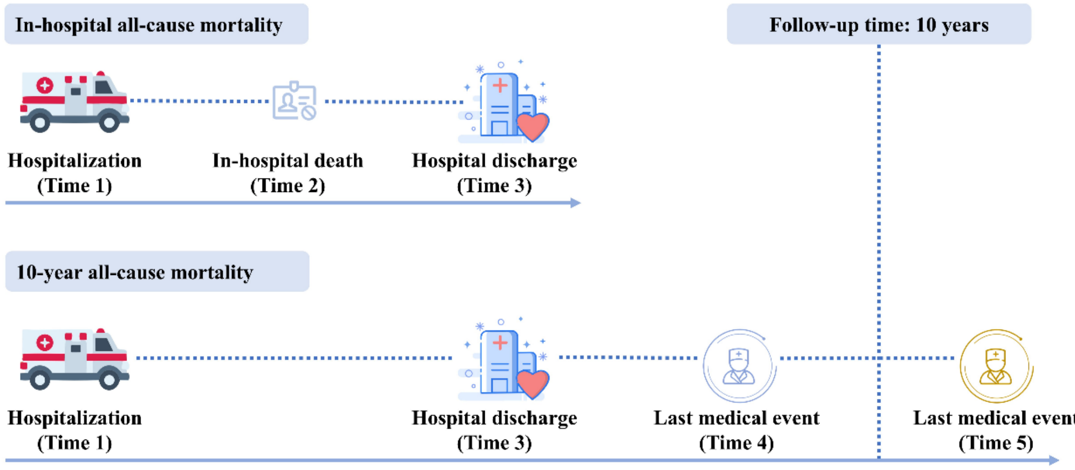

Data sources for follow-up:

TAMI cohort: Patients were followed via outpatient visits, telephone contact, and questionnaires administered by trained investigators. In-hospital mortality data were collected from the hospital information system (HIS). Out-of-hospital mortality information was obtained through follow-up inquiries made to patients or their relatives.

MIMIC-IV database: In-hospital mortality data were extracted directly from electronic health records. Out-of-hospital mortality data were obtained by linking patient identifiers with the national Social Security Administration Death Master File, which provides exact dates of death.

eICU-CRD database: In-hospital mortality data were extracted directly from electronic health records. Due to the absence of linkage with external mortality databases, data on long-term out-of-hospital mortality were unavailable.

Definitions of time variables:

Time 1: Date of CCU/ICU admission

Time 2: Date of in-hospital death

Time 3: Date of hospital discharge

Time 4: Date of last follow-up or censoring

Calculation of follow-up time:

In-hospital all-cause mortality:

Survival to discharge: Follow-up = Time 3 – Time 1 (Status = 0; alive)

In-hospital death: Follow-up = Time 2 – Time 1 (Status = 1; dead)

10-year all-cause mortality:

Survival post-discharge and follow-up time less than 10 years: Time 4 – Time 1 (Status = 0; alive)

Death post-discharge and follow-up time less than 10 years: Time 4 – Time 1 (Status = 1; dead)

Follow-up exceeding 10 years (censored at 10 years): Follow-up = 3650 days (Status = 0; alive)

Supplementary Figure 3. Directed acyclic graph.

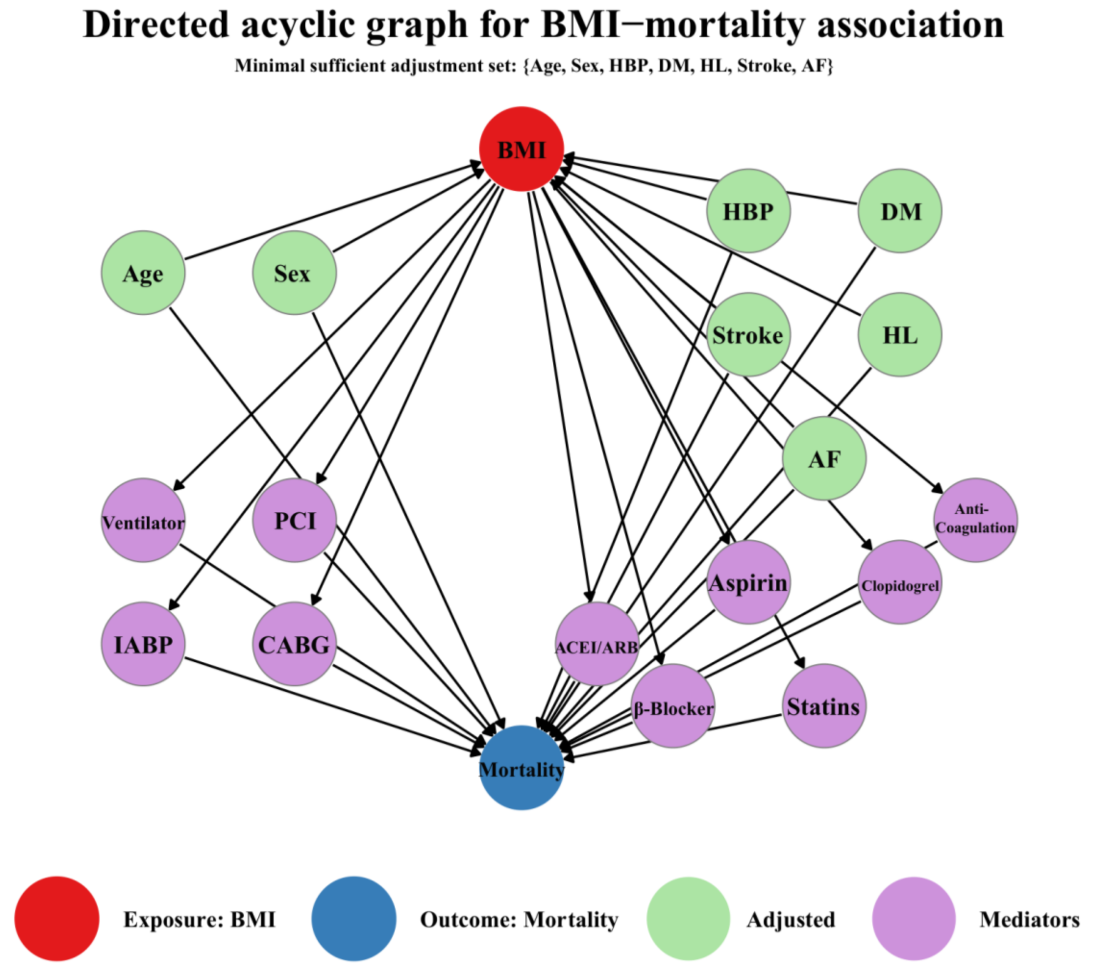

Notes: This directed acyclic graph (DAG) formalizes our causal framework. The minimal sufficient adjustment set consisted of age, sex, hypertension (HBP), diabetes mellitus (DM), hyperlipidemia (HL), stroke, and atrial fibrillation (AF). Treatments and medications were considered mediators and therefore not adjusted for in the primary analysis to avoid over-adjustment bias. HBP, hypertension; DM, diabetes mellitus; HL, hyperlipidemia; AF, atrial fibrillation; BMI, body mass index; PCI, percutaneous coronary intervention; CABG, coronary-artery-bypass-grafting; IABP, intra-aortic balloon pump; ACEI, angiotensin-converting enzyme inhibitor; ARB, angiotensin II receptor blocker; DAG, directed acyclic graph.

Supplementary Figure 4. Kaplan-Meier curves for risk of all-cause mortality in NSTEMI patients grouped by BMI combined with CRP level.

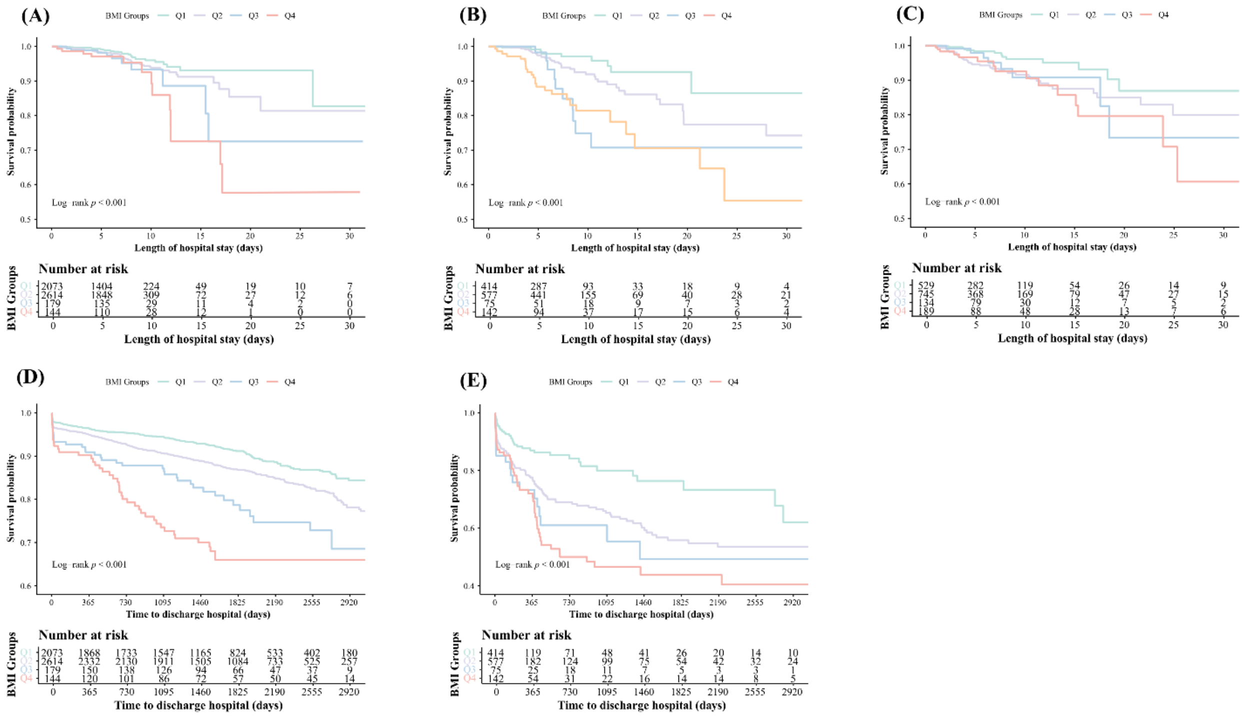

Notes: Q1: low BMI/low CRP (BMI < 35 kg/m^2 and CRP < 2 mg/L); Q2: low BMI/high CRP (BMI < 35 kg/m^2 and CRP ≥ 2 mg/L); Q3: high BMI/low CRP (BMI ≥ 35 kg/m^2 and CRP < 2 mg/L); Q4: high BMI/high CRP (BMI ≥ 35 kg/m^2 and CRP ≥ 2 mg/L). (A) In-hospital all-cause mortality in the TAMI cohort; (B) In-hospital all-cause mortality in the MIMIC-IV cohort; (C) In-hospital all-cause mortality in the eICU-CRD cohort; (D) 10-year all-cause mortality in the TAMI cohort; (E) 10-year all-cause mortality in the MIMIC-IV cohort. NSTEMI, non-ST-segment elevation myocardial infarction; BMI, body mass index; CRP, C-reactive protein.

Supplementary Figure 5. Subgroup analysis of BMI and in-hospital all-cause mortality in the three cohorts.

Variable	Event/Total	Incidence rate (%)	HR (95% CI)	*p* value	*p* for interaction
**TAMI Cohort**					
Severe obesity (≥35.0)	27/323	8.36	2.37(1.55−3.61)	<0.001	
Age					0.921
< 80	23/310	7.42	2.30(1.43−3.68)	0.001	
≥ 80	4/13	30.77	1.32(0.79−3.83)	0.126	
Sex					0.372
Male	16/415	3.86	2.75(1.56−4.82)	<0.001	
Female	11/175	6.29	2.00(1.04−3.84)	0.039	
Hypertension					0.372
Yes	24/264	9.09	2.54(1.49−4.32)	0.001	
No	3/59	5.08	1.88(0.89−3.94)	0.097	
Diabetes mellitus					0.019
Yes	19/150	12.67	2.88(1.81−4.59)	<0.001	
No	8/173	4.62	1.09(0.33−3.60)	0.888	
**MIMIC−IV Cohort**					
Severe obesity (≥35.0)	35/217	16.12	2.66(1.78−3.98)	<0.001	
Age					0.102
< 80	29/187	15.51	2.78(1.77−4.38)	<0.001	
≥ 80	6/30	20.00	1.29(0.58−2.88)	0.531	
Sex					0.691
Male	27/149	18.12	2.84(1.50−4.05)	<0.001	
Female	8/68	11.76	1.95(0.95−4.01)	0.069	
Hypertension					0.561
Yes	30/167	17.96	2.82(1.53−4.21)	<0.001	
No	5/50	10.00	1.90(0.95−3.82)	0.069	
Diabetes mellitus					0.132
Yes	15/93	16.13	3.00(1.76−5.09)	0.001	
No	20/124	16.13	1.94(1.76−3.02)	0.003	
**eICU−CRD Cohort**					
Severe obesity (≥35.0)	32/330	9.70	3.01(1.86−4.86)	<0.001	
Age					0.307
< 80	30/314	9.55	3.27(1.91−5.61)	<0.001	
≥ 80	2/16	12.50	1.67(0.37−7.57)	0.503	
Sex					0.375
Male	23/184	12.50	3.78(2.08−6.86)	<0.001	
Female	9/146	6.16	1.94(0.84−4.52)	0.122	
Hypertension					0.028
Yes	25/197	12.69	3.91(1.96−6.39)	<0.001	
No	7/133	5.26	1.63(0.68−3.89)	0.275	
Diabetes mellitus					0.019
Yes	24/129	18.61	4.09(1.97−7.26)	<0.001	
No	8/201	3.98	1.62(0.72−3.66)	0.244	

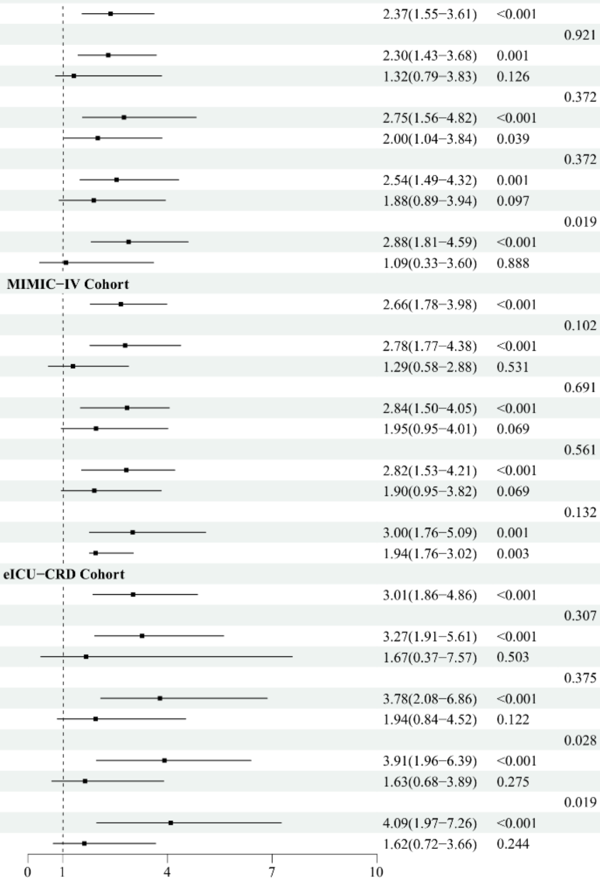

Notes: Forest plot displaying the association between severe obesity (obesity II/III vs. non-obesity II/III) and in-hospital all-cause mortality across prespecified subgroups. Hazard ratios (HRs) and 95% confidence intervals (CIs) were derived from Cox proportional hazards models (Model 3). The solid squares represent the point estimate of HR, with the size proportional to the inverse variance of the estimate. The horizontal lines indicate the 95% CI. P values for interaction were calculated by including a product term between severe obesity and each subgroup variable in the fully adjusted model. NSTEMI, non-ST-segment elevation myocardial infarction; BMI, body mass index.

Supplementary Figure 6. Subgroup analysis of BMI and 10-year all-cause mortality in the two cohorts.

Variable	Event/Total	Incidence rate (%)	HR (95% CI)	*p* value	*p* for interaction
**TAMI Cohort**					
Severe obesity (≥35.0)	90/323	27.86	2.18(1.72−2.76)	<0.001	
Age					0.309
< 80	84/310	27.11	2.33(1.82−2.99)	<0.001	
≥ 80	6/13	46.15	1.49(0.65−3.41)	0.341	
Sex					0.147
Male	93/415	22.41	2.41(1.78−3.25)	<0.001	
Female	56/175	32.00	1.82(1.26−2.65)	0.002	
Hypertension					0.025
Yes	88/264	33.33	2.45(1.92−3.11)	<0.001	
No	2/59	3.39	0.42(0.11−1.71)	0.227	
Diabetes mellitus					0.019
Yes	66/150	44.00	2.69(2.04−3.54)	<0.001	
No	24/173	13.87	1.28(0.79−2.06)	0.321	
**MIMIC−IV Cohort**					
Severe obesity (≥35.0)	73/217	33.64	2.04(1.53−2.73)	<0.001	
Age					0.148
< 80	59/187	31.55	2.31(1.64−3.26)	<0.001	
≥ 80	14/30	46.67	1.67(0.92−3.02)	0.091	
Sex					0.421
Male	52/149	34.89	2.42(1.70−3.44)	<0.001	
Female	21/68	30.88	1.55(0.91−2.64)	0.105	
Hypertension					0.649
Yes	46/167	27.54	2.22(1.40−3.53)	<0.001	
No	27/50	54.00	2.03(1.40−2.95)	0.001	
Diabetes mellitus					0.651
Yes	36/93	38.71	2.47(1.35−3.48)	0.001	
No	37/124	29.84	2.01(1.38−2.92)	<0.001	

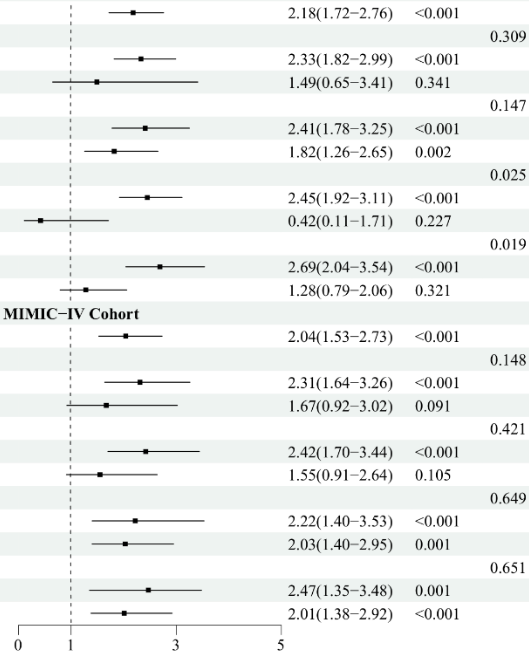

Notes: Forest plot displaying the association between severe obesity (obesity II/III vs. non-obesity II/III) and 10-year all-cause mortality across prespecified subgroups. Hazard ratios (HRs) and 95% confidence intervals (CIs) were derived from Cox proportional hazards models (Model 3). The solid squares represent the point estimate of HR, with the size proportional to the inverse variance of the estimate. The horizontal lines indicate the 95% CI. P values for interaction were calculated by including a product term between severe obesity and each subgroup variable in the fully adjusted model. NSTEMI, non-ST-segment elevation myocardial infarction; BMI, body mass index.

Supplementary Figure 7. Subgroup forest plots of BMI combined with CRP and in-hospital all-cause mortality in the three cohorts.

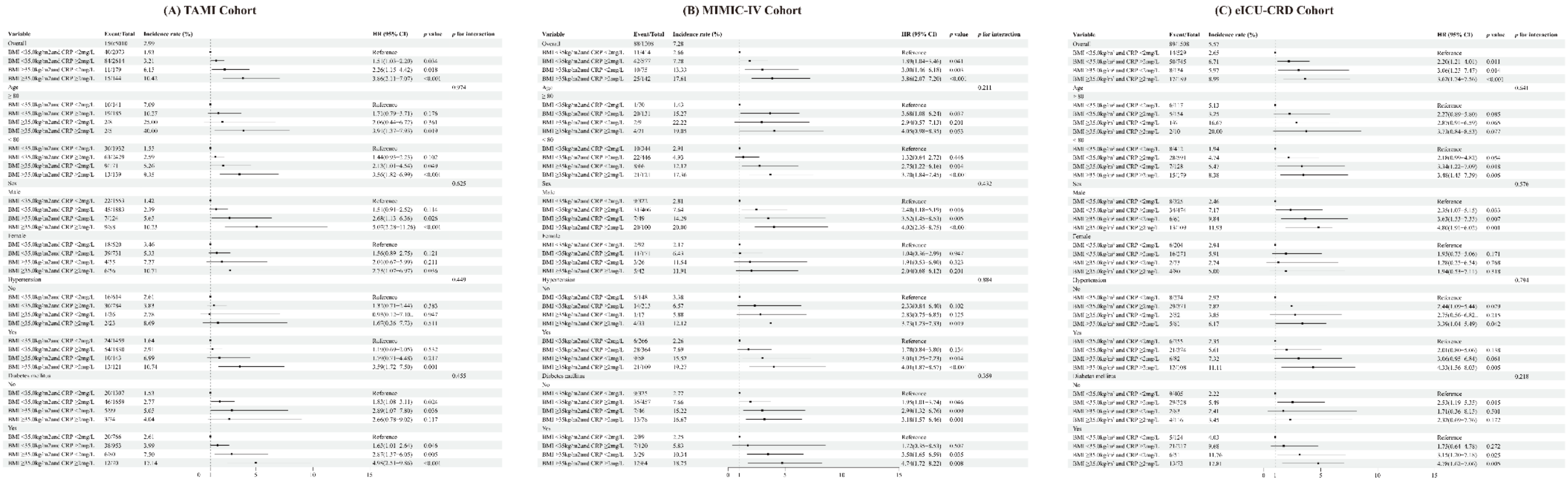

Notes: Forest plots displaying the association between the combined phenotype (severe obesity plus elevated CRP) and in-hospital all-cause mortality across prespecified subgroups. Hazard ratios (HRs) and 95% confidence intervals (CIs) were derived from Cox proportional hazards models (Model 3). The solid squares represent the point estimate of HR, with the size proportional to the inverse variance of the estimate. The horizontal lines indicate the 95% CI. P values for interaction were calculated by including a product term between severe obesity plus elevated CRP and each subgroup variable in the fully adjusted model. NSTEMI, non-ST-segment elevation myocardial infarction; BMI, body mass index.

Supplementary Figure 8. Subgroup forest plots of BMI combined with CRP and 10-year all-cause mortality in the two cohorts.

(A) TAMI Cohort

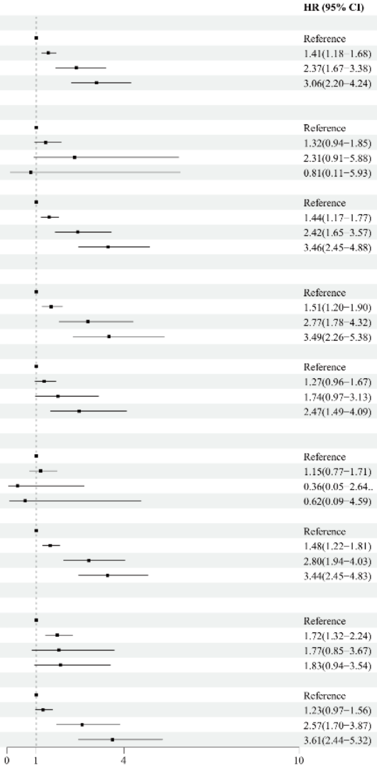

Variable	Event/Total	Incidence rate (%)	HR (95% CI)	*p* value	*p* for interaction
Overall	635/5010	12.67			
BMI <35.0kg/m2and CRP <2mg/L	190/2073	9.17	Reference		
BMI <35.0kg/m2and CRP ≥2mg/L	355/2614	13.58	1.41(1.18–1.68)	<0.001	
BMI ≥35.0kg/m2and CRP <2mg/L	37/179	20.67	2.37(1.67–3.38)	<0.001	
BMI ≥35.0kg/m2and CRP ≥2mg/L	53/144	36.81	3.06(2.20–4.24)	<0.001	
Age					0.306
≥ 80					
BMI <35.0kg/m2and CRP <2mg/L	53/141	37.59	Reference		
BMI <35.0kg/m2and CRP ≥2mg/L	91/185	49.19	1.32(0.94–1.85)	0.111	
BMI ≥35.0kg/m2and CRP <2mg/L	5/8	62.50	2.31(0.91–5.88)	0.079	
BMI ≥35.0kg/m2and CRP ≥2mg/L	1/5	20.00	0.81(0.11–5.93)	0.835	
< 80					
BMI <35.0kg/m2and CRP <2mg/L	137/1932	7.09	Reference		
BMI <35.0kg/m2and CRP ≥2mg/L	264/2429	10.87	1.44(1.17–1.77)	0.001	
BMI ≥35.0kg/m2and CRP <2mg/L	32/171	18.71	2.42(1.65–3.57)	<0.001	
BMI ≥35.0kg/m2and CRP ≥2mg/L	52/139	37.41	3.46(2.45–4.88)	<0.001	
Sex					0.391
Male					
BMI <35.0kg/m2and CRP <2mg/L	111/1553	7.15	Reference		
BMI <35.0kg/m2and CRP ≥2mg/L	214/1883	11.36	1.51(1.20–1.90)	<0.001	
BMI ≥35.0kg/m2and CRP <2mg/L	24/124	19.35	2.77(1.78–4.32)	<0.001	
BMI ≥35.0kg/m2and CRP ≥2mg/L	30/88	34.09	3.49(2.26–5.38)	<0.001	
Female					
BMI <35.0kg/m2and CRP <2mg/L	79/520	15.19	Reference		
BMI <35.0kg/m2and CRP ≥2mg/L	141/731	19.29	1.27(0.96–1.67)	0.092	
BMI ≥35.0kg/m2and CRP <2mg/L	13/55	23.64	1.74(0.97–3.13)	0.065	
BMI ≥35.0kg/m2and CRP ≥2mg/L	23/56	41.07	2.47(1.49–4.09)	<0.001	
Hypertension					0.027
No					
BMI <35.0kg/m2and CRP <2mg/L	39/614	6.35	Reference		
BMI <35.0kg/m2and CRP ≥2mg/L	66/784	8.42	1.15(0.77–1.71)	0.501	
BMI ≥35.0kg/m2and CRP <2mg/L	1/36	2.78	0.36(0.05–2.64..	0.315	
BMI ≥35.0kg/m2and CRP ≥2mg/L	1/23	4.35	0.62(0.09–4.59)	0.644	
Yes					
BMI <35.0kg/m2and CRP <2mg/L	151/1459	10.35	Reference		
BMI <35.0kg/m2and CRP ≥2mg/L	289/1830	15.79	1.48(1.22–1.81)	<0.001	
BMI ≥35.0kg/m2and CRP <2mg/L	36/143	25.17	2.80(1.94–4.03)	<0.001	
BMI ≥35.0kg/m2and CRP ≥2mg/L	52/121	42.98	3.44(2.45–4.83)	<0.001	
Diabetes mellitus					0.017
No					
BMI <35.0kg/m2and CRP <2mg/L	79/1307	6.04	Reference		
BMI <35.0kg/m2and CRP ≥2mg/L	180/1659	10.85	1.72(1.32–2.24)	<0.001	
BMI ≥35.0kg/m2and CRP <2mg/L	8/99	8.08	1.77(0.85–3.67)	0.124	
BMI ≥35.0kg/m2and CRP ≥2mg/L	16/74	21.62	1.83(0.94–3.54)	0.074	
Yes					
BMI <35.0kg/m2and CRP <2mg/L	111/766	14.49	Reference		
BMI <35.0kg/m2and CRP ≥2mg/L	175/953	18.32	1.23(0.97–1.56)	0.094	
BMI ≥35.0kg/m2and CRP <2mg/L	29/80	36.25	2.57(1.70–3.87)	<0.001	
BMI ≥35.0kg/m2and CRP ≥2mg/L	37/70	52.86	3.61(2.44–5.32)	<0.001	

(B) MIMIC-IV Cohort

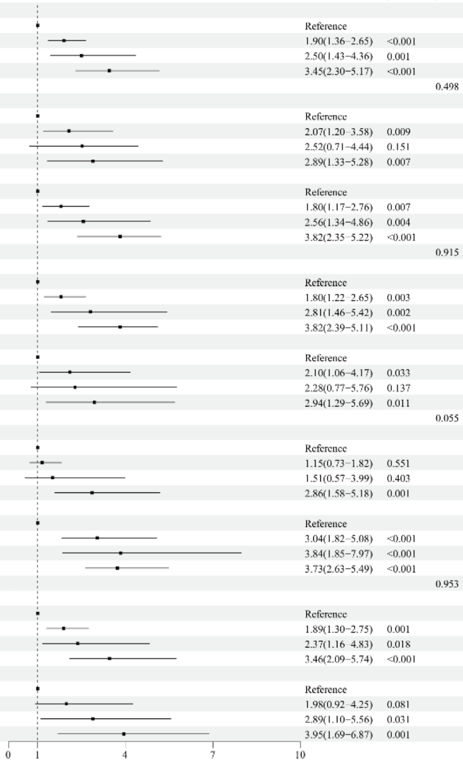

Variable	Event/Total	Incidence rate (%)	HR (95% CI)	*p* value	*p* for interaction
Overall	260/1208	21.52			
BMI <35kg/m^2 and CRP <2mg/L	46/414	11.11	Reference		
BMI <35kg/m^2 and CRP ≥2mg/L	141/577	24.44	1.90(1.36–2.65)	<0.001	
BMI ≥35kg/m^2 and CRP <2mg/L	22/75	29.33	2.50(1.43–4.36)	0.001	
BMI ≥35kg/m^2 and CRP ≥2mg/L	51/142	35.92	3.45(2.30–5.17)	<0.001	
Age					0.498
≥ 80					
BMI <35kg/m^2 and CRP <2mg/L	17/70	24.29	Reference		
BMI <35kg/m^2 and CRP ≥2mg/L	60/131	45.79	2.07(1.20–3.58)	0.009	
BMI ≥35kg/m^2 and CRP <2mg/L	3/9	33.33	2.52(0.71–4.44)	0.151	
BMI ≥35kg/m^2 and CRP ≥2mg/L	11/21	52.38	2.89(1.33–5.28)	0.007	
< 80					
BMI <35kg/m^2 and CRP <2mg/L	29/344	8.43	Reference		
BMI <35kg/m^2 and CRP ≥2mg/L	81/446	18.16	1.80(1.17–2.76)	0.007	
BMI ≥35kg/m^2 and CRP <2mg/L	19/66	28.79	2.56(1.34–4.86)	0.004	
BMI ≥35kg/m^2 and CRP ≥2mg/L	40/121	33.06	3.82(2.35–5.22)	<0.001	
Sex					0.915
Male					
BMI <35kg/m^2 and CRP <2mg/L	36/322	11.18	Reference		
BMI <35kg/m^2 and CRP ≥2mg/L	90/406	22.17	1.80(1.22–2.65)	0.003	
BMI ≥35kg/m^2 and CRP <2mg/L	16/49	32.65	2.81(1.46–5.42)	0.002	
BMI ≥35kg/m^2 and CRP ≥2mg/L	36/100	36.00	3.82(2.39–5.11)	<0.001	
Female					
BMI <35kg/m^2 and CRP <2mg/L	10/92	10.87	Reference		
BMI <35kg/m^2 and CRP ≥2mg/L	51/171	29.82	2.10(1.06–4.17)	0.033	
BMI ≥35kg/m^2 and CRP <2mg/L	6/26	23.08	2.28(0.77–5.76)	0.137	
BMI ≥35kg/m^2 and CRP ≥2mg/L	15/42	35.71	2.94(1.29–5.69)	0.011	
Hypertension					0.055
No					
BMI <35kg/m^2 and CRP <2mg/L	28/148	18.92	Reference		
BMI <35kg/m^2 and CRP ≥2mg/L	61/213	28.64	1.15(0.73–1.82)	0.551	
BMI ≥35kg/m^2 and CRP <2mg/L	6/17	35.29	1.51(0.57–3.99)	0.403	
BMI ≥35kg/m^2 and CRP ≥2mg/L	20/33	60.61	2.86(1.58–5.18)	0.001	
Yes					
BMI <35kg/m^2 and CRP <2mg/L	18/266	6.77	Reference		
BMI <35kg/m^2 and CRP ≥2mg/L	80/364	21.98	3.04(1.82–5.08)	<0.001	
BMI ≥35kg/m^2 and CRP <2mg/L	16/58	27.59	3.84(1.85–7.97)	<0.001	
BMI ≥35kg/m^2 and CRP ≥2mg/L	31/109	28.44	3.73(2.63–5.49)	<0.001	
Diabetes mellitus					0.953
No					
BMI <35kg/m^2 and CRP <2mg/L	37/325	11.38	Reference		
BMI <35kg/m^2 and CRP ≥2mg/L	113/457	24.73	1.89(1.30–2.75)	0.001	
BMI ≥35kg/m^2 and CRP <2mg/L	12/46	26.09	2.37(1.16–4.83)	0.018	
BMI ≥35kg/m^2 and CRP ≥2mg/L	25/78	32.05	3.46(2.09–5.74)	<0.001	
Yes					
BMI <35kg/m^2 and CRP <2mg/L	9/89	10.11	Reference		
BMI <35kg/m^2 and CRP ≥2mg/L	28/120	23.33	1.98(0.92–4.25)	0.081	
BMI ≥35kg/m^2 and CRP <2mg/L	10/29	34.48	2.89(1.10–5.56)	0.031	
BMI ≥35kg/m^2 and CRP ≥2mg/L	26/64	40.63	3.95(1.69–6.87)	0.001	

Notes: Forest plots displaying the association between the combined phenotype (severe obesity plus elevated CRP) and 10-year all-cause mortality across prespecified subgroups. Hazard ratios (HRs) and 95% confidence intervals (CIs) were derived from Cox proportional hazards models (Model 3). The solid squares represent the point estimate of HR, with the size proportional to the inverse variance of the estimate. The horizontal lines indicate the 95% CI. P values for interaction were calculated by including a product term between severe obesity plus elevated CRP and each subgroup variable in the fully adjusted model. NSTEMI, non-ST-segment elevation myocardial infarction; BMI, body mass index.

Supplementary Figure 9. Calibration curves comparing the base model and the incremental model with CRP for predicting all-cause mortality.

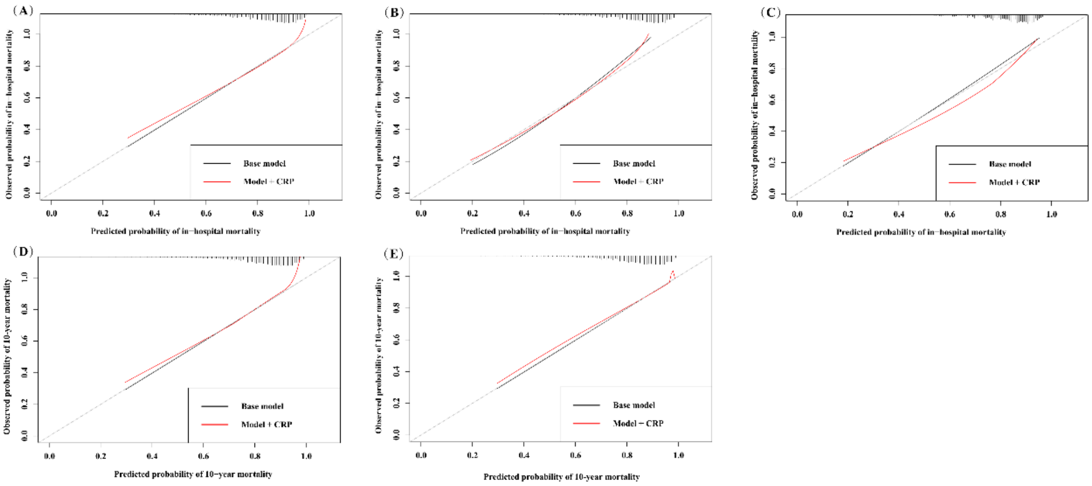

Notes: Calibration curves were generated using 1,000 bootstrap resamples with the rms package. (A) TAMI cohort, in-hospital all-cause mortality; (B) MIMIC-IV cohort, in-hospital all-cause mortality; (C) eICU-CRD cohort, in-hospital all-cause mortality; (D) TAMI cohort, 10-year all-cause mortality; (E) MIMIC-IV cohort, 10-year all-cause mortality. The black curve represents the base model (adjusted for age, sex, hypertension, diabetes mellitus, hyperlipidemia, stroke, atrial fibrillation, and BMI categories). The red curve represents the incremental model (base model + CRP). Both curves closely follow the diagonal reference line, indicating good calibration. The calibration slope was close to 1.0 and the maximum calibration error (Emax) was < 0.05 for both models in all cohorts, suggesting no significant difference in calibration after adding CRP. CRP, C-reactive protein; BMI, body mass index.

Supplementary Figure 10. Mediation analyses of CRP in the association between severe obesity and all-cause mortality.

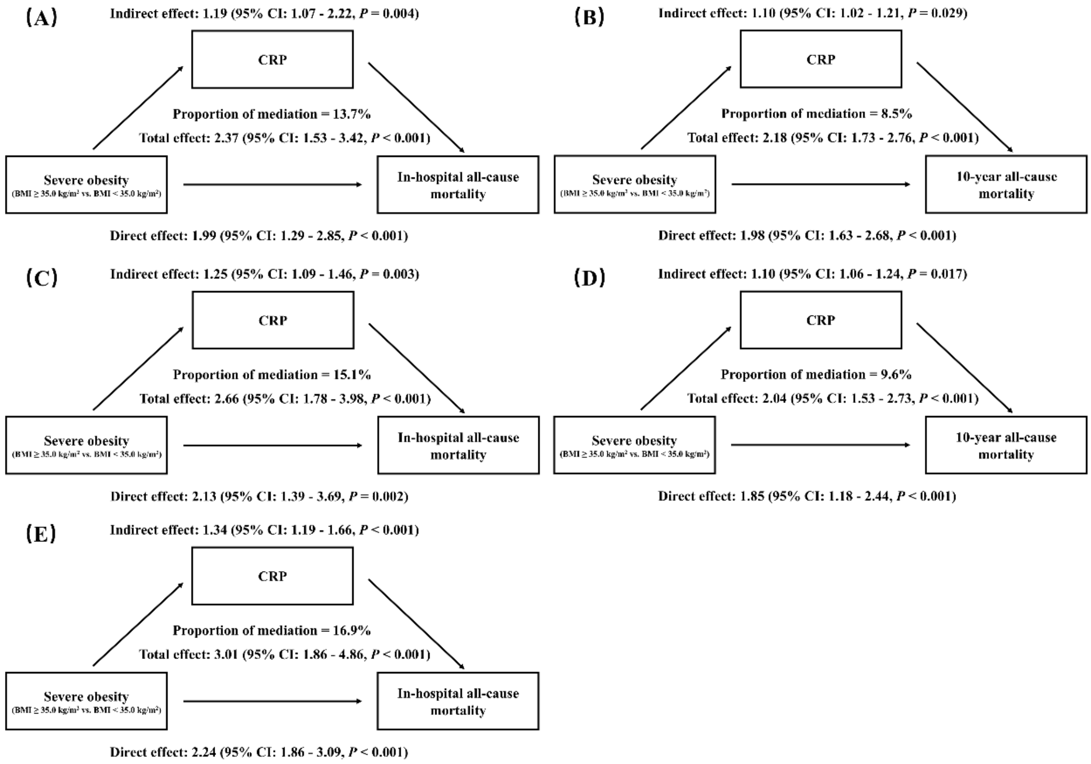

Notes: Mediation analyses were performed using the fully adjusted model (Model 3) with 1,000 bootstrap resamples. (A) TAMI cohort, in-hospital all-cause mortality; (B) TAMI cohort, 10-year all-cause mortality; (C) MIMIC-IV cohort, in-hospital all-cause mortality; (D) MIMIC-IV cohort, 10-year all-cause mortality; (E) eICU-CRD cohort, in-hospital all-cause mortality. The total effect, direct effect, and indirect effect are presented as hazard ratios (HR) with 95% confidence intervals (CI). The proportion of mediation represents the percentage of the total effect explained by CRP. All *p* values are two-sided. CRP, C-reactive protein; BMI, body mass index; HR, hazard ratio; CI, confidence interval.

Supplementary Figure 11. Meta-analysis of BMI categories and all-cause mortality across the three cohorts.

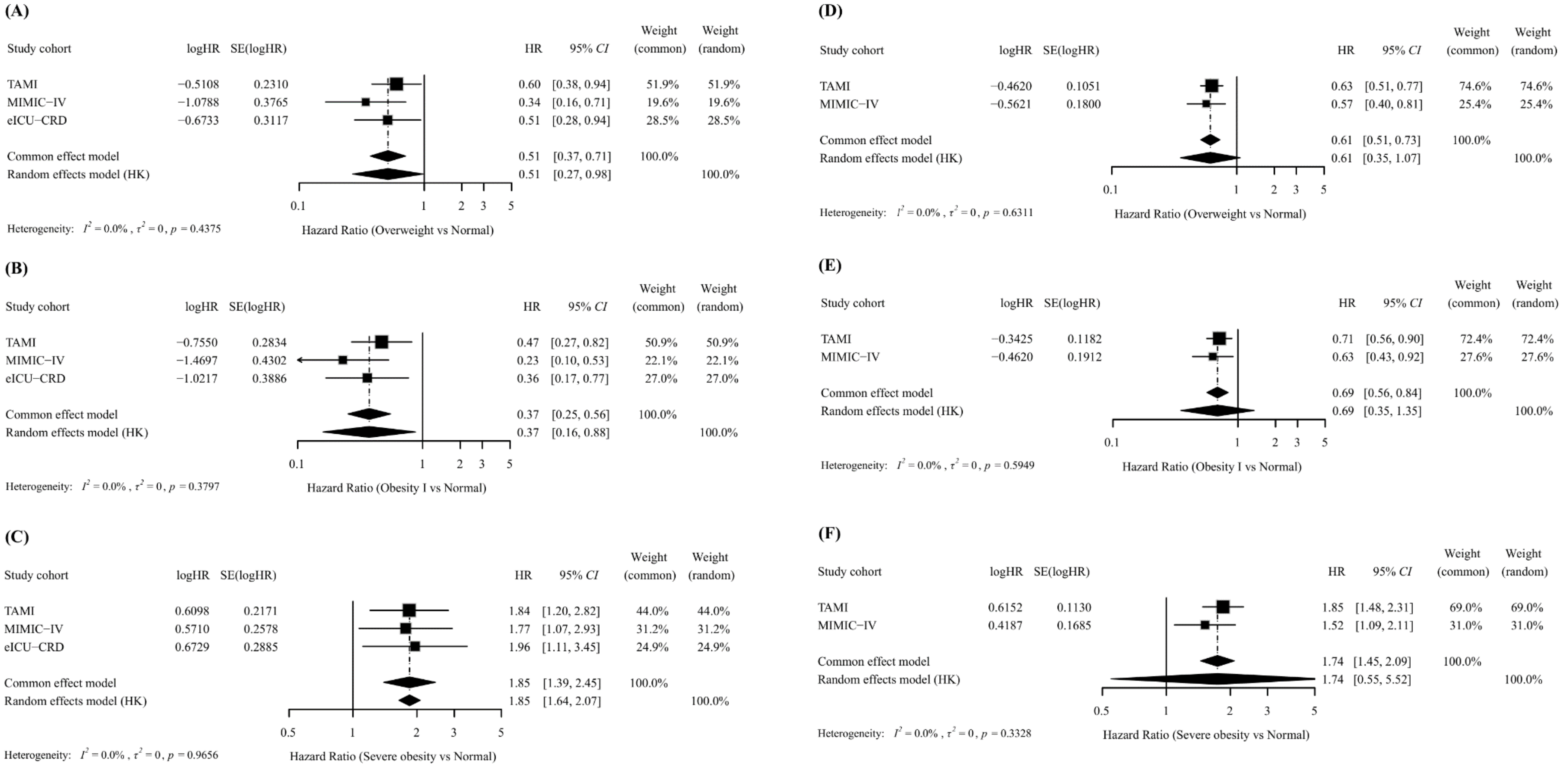

Notes: Random-effects models with Hartung-Knapp adjustment were used to pool the hazard ratios (HRs) from the three cohorts. (A) In-hospital mortality, overweight vs. normal; (B) In-hospital mortality, obesity I vs. normal; (C) In-hospital mortality, severe obesity vs. normal; (D) 10-year mortality, overweight vs. normal; (E) 10-year mortality, obesity I vs. normal; (F) 10-year mortality, severe obesity vs. normal. Heterogeneity was assessed using the I² statistic. BMI, body mass index; HR, hazard ratio.

Supplementary Table 1. Detailed information of three cohorts.

Tianjin Inpatient Acute Myocardial Infarction (TAMI) cohort:

Acute myocardial infarction (AMI) is the most serious manifestation of coronary artery disease. AMI is characterized by high mortality, high disability rates, and high cost. However, large-scale multicenter research on AMI in Tianjin or even China is limited. By including AMI in Tianjin Chest Hospital, this single-center study captures the changes in epidemiological trends, analyze treatment patterns in Tianjin, and explore optimal treatment strategies. Since January 2015, the registry has consecutively enrolled all patients admitted with AMI. Comprehensive in-hospital data are extracted from the hospital's electronic health record system, encompassing vital signs, laboratory test results, medication administration, fluid balance, diagnosis codes, billing records, and details of clinical interventions such as mechanical ventilation and invasive procedures. To ensure longitudinal follow-up, trained cardiologists conduct structured post-discharge assessments via outpatient visits, hospital readmission records, and telephone interviews. This design supports analyses of both in-hospital and long-term outcomes, making the TAMI database a valuable resource for outcome prediction, phenotyping studies, and epidemiological research in critical cardiovascular care. The registry was approved by the Ethics Committee of Tianjin Chest Hospital (approval number: 2018KY-010-01). Written informed consent was obtained from all participating patients, and the study was conducted in strict accordance with the principles of the Declaration of Helsinki.

Medical Information Mart for Intensive Care IV (MIMIC-IV):

MIMIC-IV is a large, single-center, and publicly available database comprising de-identified health-related data associated with over 70,000 patients admitted to critical care units at the Beth Israel Deaconess Medical Center between 2008 and 2019. The database integrates a wide range of high-resolution, timestamped clinical data, including vital signs, laboratory measurements, medication administration, fluid balance, diagnostic codes, and physician notes. It also contains comprehensive hospital billing diagnoses and detailed information on clinical interventions such as ventilator management, renal replacement therapy, and invasive procedures. These granular, high-frequency data enable the validation and enrichment of primary diagnoses and can reveal clinical trajectories not fully ascertainable from coded data alone. As a result of its detailed clinical data, MIMIC-IV supports longitudinal analyses of in-hospital outcomes and has been extensively utilized for outcome prediction, phenotyping, and epidemiological research in critical care. Ethical approval for the MIMIC-IV database was granted by the Institutional Review Boards of the Massachusetts Institute of Technology and Beth Israel Deaconess Medical Center, with a waiver for informed consent due to the use of de-identified data.

eICU Collaborative Research Database (eICU-CRD):

The eICU-CRD is a multi-center, publicly available database comprising de-identified health data of more than 200,000 patients admitted to intensive care units across the United States between 2014 and 2015. The database aggregates clinical information from over 200 hospitals, encompassing vital signs, laboratory results, medication records, treatment plans, nursing assessments, and diagnosis codes. It further includes detailed documentation on organ dysfunction scores, ventilator management, and other critical care interventions. These highly granular, multi-source data facilitate external validation of clinical phenotypes and allow exploration of care patterns and outcomes across diverse healthcare settings. As a result of its geographic and institutional diversity, the eICU-CRD supports investigations into variations in critical care practice and research aimed at understanding systemic and patient-level determinants of ICU outcomes. Ethical approval for the use of the eICU-CRD was obtained from the Massachusetts Institute of Technology, with individual patient consent waived due to the retrospective and de-identified nature of the data.

Supplementary Table 2. NSTEMI diagnostic criteria in three cohorts.

TAMI cohort:

The diagnosis of NSTEMI was based on the Fourth Universal Definition of Myocardial Infarction, as follows: Detection of a rise and/or fall of cTnT/cTnI values with at least 1 value above the 99th percentile URL and with at least 1 of the following:

(1) Symptoms of acute myocardial ischemia;
(2) New ischemic ECG changes;
(3) Development of pathological Q waves;
(4) Imaging evidence of new loss of viable myocardium or new regional wall motion abnormality in a pattern consistent with an ischemic etiology;
(5) Identification of a coronary thrombus by angiography including intracoronary imaging or by autopsy;
(6) No ST-segment elevation on ECG.

Patients who were diagnosed with NSTEMI at discharge were extracted from the electronic medical record system. Two independent cardiologists reviewed the electrocardiogram data, laboratory examination and coronary angiography.

Medical Information Mart for Intensive Care IV (MIMIC-IV) cohort:

The MIMIC-IV database was extracted based on the ICD-9 and ICD-10 diagnostic codes for NSTEMI, as follows:

(1) ICD-9: 410.70, 410.71, 410.72;
(2) ICD-10: I21.4.

eICU Collaborative Research Database (eICU-CRD):

The eICU-CRD database was extracted based on the ICD-9 diagnostic codes for NSTEMI, as follows:

(1) ICD-9: 410.70, 410.71, 410.72.

Supplementary Table 3. BMI groups in three cohorts.

In the main analysis, all three cohorts were grouped according to the World Health Organization (WHO) obesity criteria. For the TAMI cohort, we additionally applied the Chinese obesity guidelines to define BMI categories as a sensitivity analysis to test the robustness of the main findings.

The specific BMI categories were as follows:

TAMI Cohort:

WHO criteria:

Normal: 18.5-24.9 kg/m^2

Overweight: 25.0-29.9 kg/m^2

Obesity I: 30.0-34.9 kg/m^2

Severe obesity: ⩾35.0 kg/m^2

Chinese criteria:

Normal: 18.5-23.9 kg/m^2

Overweight: 24.0-27.9 kg/m^2

Obesity I: 28.0-32.5 kg/m^2

Severe obesity: ≥32.5 kg/m^2

Medical Information Mart for Intensive Care IV (MIMIC-IV) cohort:

WHO criteria:

Normal: 18.5-24.9 kg/m^2

Overweight: 25.0-29.9 kg/m^2

Obesity I: 30.0-34.9 kg/m^2

Severe obesity: ⩾35.0 kg/m^2

eICU Collaborative Research Database (eICU-CRD) cohort:

WHO criteria:

Normal: 18.5-24.9 kg/m^2

Overweight: 25.0-29.9 kg/m^2

Obesity I: 30.0-34.9 kg/m^2

Severe obesity: ⩾35.0 kg/m^2

Supplementary Table 4. Schoenfeld residual tests for proportional hazards assumption.

Variable	TAMI cohort				MIMIC-IV cohort				eICU-CRD cohort	
	In-hospital		10-year		In-hospital		10-year		In-hospital	
	χ^2	*p* value	χ^2	*p* value	χ^2	*p* value	χ^2	*p* value	χ^2	*p* value
BMI (category)	7.65	0.054	5.72	0.126	1.11	0.775	5.45	0.142	2.15	0.543
Age	2.32	0.127	3.57	0.059	0.29	0.588	1.53	0.216	0.79	0.381
Sex	0.23	0.635	1.46	0.227	3.12	0.077	0.08	0.781	0.59	0.444
Hypertension	0.86	0.355	2.95	0.086	0.29	0.591	4.83	0.081	1.81	0.183
Diabetes mellitus	0.03	0.856	0.52	0.471	0.11	0.753	0.57	0.451	1.07	0.302
Hyperlipidemia	0.04	0.861	0.99	0.318	0.06	0.809	0.16	0.689	0.93	0.347
Stroke	2.09	0.147	0.04	0.851	1.54	0.215	0.03	0.855	0.53	0.477
Atrial fibrillation	0.04	0.847	0.07	0.792	2.68	0.101	0.16	0.687	0.53	0.477
Global	15.19	0.125	13.85	0.181	9.26	0.508	13.44	0.201	4.85	0.906

Notes: Schoenfeld residual tests were performed for each covariate in the final Cox proportional hazards models (Model 3). A p value > 0.05 indicates no violation of the proportional hazards assumption. All global tests and individual covariate tests yielded p values > 0.05, confirming that the proportional hazards assumption was satisfied across all models. The eICU-CRD database lacks long-term follow-up data; therefore, only in-hospital analyses were performed. χ^2, Chi-squared statistic; BMI, body mass index; TAMI, Tianjin Inpatient Acute Myocardial Infarction; MIMIC-IV, Medical Information Mart for Intensive Care IV; eICU-CRD, eICU Collaborative Research Database.

Supplementary Table 5. The variance inflation factor of covariates in the three cohorts.

Characteristics	TAMI cohort	MIMIC-IV cohort	eICU-CRD cohort
Age	1.07	1.04	1.12
Sex	1.04	1.03	1.03
Hypertension	1.05	1.04	1.06
Diabetes mellitus	1.05	1.07	1.04
Hyperlipidemia	1.17	1.02	1.13
Atrial fibrillation	1.03	1.02	1.05
Stroke	1.03	1.02	1.07

Notes: To assess multicollinearity, we calculated variance inflation factors (VIFs) for all covariates. All VIF values were below 2, which is well under the commonly accepted threshold of 5 (or 10), indicating no substantial multicollinearity among the predictor variables. VIF, variance inflation factor.

Supplementary Table 6. Missing values of variables.

Characteristics	TAMI cohort		MIMIC-IV cohort		eICU-CRD cohort	
	Missing count	Missing percentage	Missing count	Missing percentage	Missing count	Missing percentage
**Demographic characteristics**						
Age	0	0.00%	0	0.00%	0	0.00%
Sex	0	0.00%	0	0.00%	0	0.00%
Weight	0	0.00%	0	0.00%	0	0.00%
Height	0	0.00%	0	0.00%	0	0.00%
BMI	0	0.00%	0	0.00%	0	0.00%
**Comorbidity**						
Hypertension	0	0.00%	0	0.00%	0	0.00%
Diabetes mellitus	0	0.00%	0	0.00%	0	0.00%
Hyperlipidemia	0	0.00%	0	0.00%	0	0.00%
Atrial fibrillation	0	0.00%	0	0.00%	0	0.00%
Stroke	0	0.00%	0	0.00%	0	0.00%
**Laboratory indexes**						
cTnT	254	5.07%	209	17.31%	230	14.41%
CK	205	4.09%	198	16.39%	142	8.89%
CK-MB	205	4.09%	198	16.39%	144	9.02%
CRP	314	6.27%	239	19.78%	367	22.98%
WBC	221	4.41%	21	1.74%	111	6.95%
Albumin	103	2.06%	98	8.11%	125	7.83%
Creatinine	461	9.20%	67	5.55%	83	5.19%
Urea nitrogen	469	9.36%	72	5.96%	92	5.76%
LDL-C	442	8.82%	225	18.63%	306	19.16%
HDL-C	443	8.84%	225	18.63%	286	17.91%
**Treatment**						
Ventilator	0	0.00%	0	0.00%	0	0.00%
IABP	0	0.00%	0	0.00%	0	0.00%
PCI	0	0.00%	0	0.00%	0	0.00%
CABG	0	0.00%	0	0.00%	0	0.00%
**Medicine application**						
Aspirin	0	0.00%	0	0.00%	0	0.00%
Clopidogrel	0	0.00%	0	0.00%	0	0.00%
Statins	0	0.00%	0	0.00%	0	0.00%
β-Blockers	0	0.00%	0	0.00%	0	0.00%
Anticoagulation	0	0.00%	0	0.00%	0	0.00%
ACEI/ARB	0	0.00%	0	0.00%	0	0.00%

Notes: cTnT, cardiac troponin T; CK, creatine kinase; CK-MB, creatine kinase MB; CRP, C-reactive protein; WBC, white blood cell; LDL-C, low-density lipoprotein cholesterol; HDL-C, high-density lipoprotein cholesterol; IABP, intra-aortic balloon pump; PCI, percutaneous coronary intervention; CABG, coronary-artery-bypass-grafting; ACEI, angiotensin-converting enzyme inhibitor; ARB, angiotensin II receptor blocker.

Supplementary Table 7. Baseline characteristics of all NSTEMI patients with different BMI levels in the MIMIC-IV cohort.

Characteristics	Overall (n = 1,208)	Normal (18.5-24.9) (n = 421)	Overweight (25.0-29.9) (n = 300)	Obesity I (30.0-34.9) (n = 270)	Severe obesity (≥35.0) (n = 217)	*p* value
**Anthropometric and demographic characteristics**						
Age, years	69.82 ± 11.09	70.09 ± 11.00	70.97 ± 11.13	70.14 ± 10.63	67.32 ± 11.45	<0.001
Sex, n (%)						0.486
Female	331 (27.40)	116 (27.55)	77 (25.67)	70 (25.93)	68 (31.34)	
Male	877 (72.60)	305 (72.45)	223 (74.33)	200 (74.07)	149 (68.66)	
Weight, kg	84.90 ± 22.05	66.22 ± 8.31	80.53 ± 9.85	94.32 ± 11.94	115.49 ± 22.65	<0.001
Height, cm	170.25 ± 9.96	171.09 ± 9.37	170.31 ± 9.49	170.85 ± 10.03	167.81 ± 11.20	<0.001
BMI, kg/m^2	29.28 ± 7.19	22.56 ± 1.49	27.68 ± 1.47	32.20 ± 1.42	40.93 ± 6.36	<0.001
**Comorbidities**						
Hypertension, n (%)						<0.001
No	411 (34.02)	148 (35.15)	110 (36.67)	103 (38.15)	50 (23.04)	
Yes	797 (65.98)	273 (64.85)	190 (63.33)	167 (61.85)	167 (76.96)	
Diabetes mellitus, n (%)						<0.001
No	906 (75.00)	334 (79.33)	237 (79.00)	211 (78.15)	124 (57.14)	
Yes	302 (25.00)	87 (20.67)	63 (21.00)	59 (21.85)	93 (42.86)	
Hyperlipidemia, n (%)						<0.001
No	1,051 (87.00)	375 (89.07)	267 (89.00)	240 (88.89)	169 (77.88)	
Yes	157 (13.00)	46 (10.93)	33 (11.00)	30 (11.11)	48 (22.12)	
Atrial fibrillation, n (%)						0.021
No	1,097 (90.81)	388 (92.16)	275 (91.67)	249 (92.22)	185 (85.25)	
Yes	111 (9.19)	33 (7.84)	25 (8.33)	21 (7.78)	32 (14.75)	
Stroke, n (%)						<0.001

No	1,031 (85.35)	365 (86.70)	263 (87.67)	235 (87.04)	168 (77.42)	
Yes	177 (14.65)	56 (13.30)	37 (12.33)	35 (12.96)	49 (22.58)	
**Laboratory parameters**						
Cardiac troponin T, ng/mL	0.29 (0.07,1.05)	0.32 (0.08,1.14)	0.26 (0.07,1.05)	0.33 (0.08,0.95)	0.24 (0.07,0.94)	0.781
Creatine kinase, U/L	167.44 (90.79,332.38)	175.30 (101.71,342.33)	161.82 (82.24,300.00)	173.09 (90.00,297.65)	153.00 (92.08,414.34)	0.571
Creatine kinase MB, U/L	21.33 (19.00,34.38)	22.00 (19.00,35.80)	21.00 (18.81,34.63)	21.33 (18.96,32.94)	21.14 (18.78,34.00)	0.784
C-reactive protein, mg/L	4.40 (1.30,13.50)	3.50 (1.30,13.40)	3.80 (1.30,11.73)	5.60 (1.33,13.68)	7.30 (1.40,15.20)	0.183
White blood cell, 10^9/L	10.95 (9.02,13.18)	10.55 (8.53,12.90)	10.49 (8.88,13.07)	11.05 (9.37,13.46)	11.74 (9.99,14.11)	<0.001
Albumin, g/L	41.70 (39.20,44.10)	40.80 (39.10,43.20)	42.60 (41.20,45.30)	42.30 (40.30,44.60)	38.90 (35.90,41.40)	<0.001
Creatinine, umol/L	96.16 (76.61,143.37)	90.26 (72.19,137.51)	98.38 (78.30,135.89)	99.81 (79.09,155.61)	101.66 (80.60,165.12)	0.028
Urea nitrogen, mmol/L	8.07 (5.91,12.60)	7.93 (5.84,12.04)	8.03 (5.71,12.17)	7.91 (5.87,12.43)	9.10 (6.37,13.66)	0.035
LDL-C, mmol/L	2.51 (2.03,3.00)	2.47 (1.97,2.96)	2.42 (1.94,2.87)	2.42 (1.91,2.85)	2.86 (2.36,3.54)	<0.001
HDL-C, mmol/L	1.20 (0.92,1.42)	1.22 (0.96,1.44)	1.22 (0.98,1.45)	1.19 (0.93,1.42)	1.10 (0.80,1.35)	<0.001
**Treatment**						
Ventilator, n (%)						<0.001
No	1,137 (94.12)	406 (96.44)	284 (94.67)	252 (93.33)	195 (89.86)	
Yes	71 (5.88)	15 (3.56)	16 (5.33)	18 (6.67)	22 (10.14)	
Intra-aortic balloon pump, n (%)						0.051
No	1,122 (92.88)	399 (94.77)	281 (93.67)	249 (92.22)	193 (88.94)	
Yes	86 (7.12)	22 (5.23)	19 (6.33)	21 (7.78)	24 (11.06)	
Percutaneous coronary intervention, n (%)						0.043
No	937 (77.57)	327 (77.67)	235 (78.33)	195 (72.22)	180 (82.95)	
Yes	271 (22.43)	94 (22.33)	65 (21.67)	75 (27.78)	37 (17.05)	
Coronary-artery-bypass-grafting, n (%)						0.017
No	1,092 (90.40)	382 (90.74)	268 (89.33)	235 (87.04)	207 (95.39)	

Yes	116 (9.60)	39 (9.26)	32 (10.67)	35 (12.96)	10 (4.61)	
**Medication use**						
Aspirin, n (%)						<0.001
No	287 (23.76)	95 (22.57)	75 (25.00)	82 (30.37)	35 (16.13)	
Yes	921 (76.24)	326 (77.43)	225 (75.00)	188 (69.63)	182 (83.87)	
Clopidogrel, n (%)						<0.001
No	733 (60.68)	271 (64.37)	188 (62.67)	170 (62.96)	104 (47.93)	
Yes	475 (39.32)	150 (35.63)	112 (37.33)	100 (37.04)	113 (52.07)	
Statins, n (%)						0.036
No	356 (29.47)	114 (27.08)	102 (34.00)	88 (32.59)	52 (23.96)	
Yes	852 (70.53)	307 (72.92)	198 (66.00)	182 (67.41)	165 (76.04)	
β-Blockers, n (%)						<0.001
No	621 (51.41)	235 (55.82)	156 (52.00)	142 (52.59)	88 (40.55)	
Yes	587 (48.59)	186 (44.18)	144 (48.00)	128 (47.41)	129 (59.45)	
Anticoagulation, n (%)						<0.001
No	326 (26.99)	118 (28.03)	94 (31.33)	78 (28.89)	36 (16.59)	
Yes	882 (73.01)	303 (71.97)	206 (68.67)	192 (71.11)	181 (83.41)	
ACEI/ARB, n (%)						<0.001
No	511 (42.30)	191 (45.37)	135 (45.00)	115 (42.59)	70 (32.26)	
Yes	697 (57.70)	230 (54.63)	165 (55.00)	155 (57.41)	147 (67.74)	
**Outcomes**						
**In-hospital all-cause mortality**						<0.001
Alive	1,120 (92.72)	383 (90.97)	291 (97.00)	264 (97.78)	182 (83.87)	
Death	88 (7.28)	38 (9.03)	9 (3.00)	6 (2.22)	35 (16.13)	
**10-year all-cause mortality**						<0.001

Alive	948 (78.48)	322 (76.48)	254 (84.67)	228 (84.44)	144 (66.36)	
Death	260 (21.52)	99 (23.52)	46 (15.33)	42 (15.56)	73 (33.64)	

Notes: Continuous variables are presented as mean ± SD if normally distributed, and median (interquartile range) if not normally distributed. Categorical variables are presented as number of patients (%). LDL-C, low-density lipoprotein cholesterol; HDL-C, high-density lipoprotein cholesterol; ACEI, angiotensin-converting enzyme inhibitor; ARB, angiotensin II receptor blocker; NSTEMI, non-ST-segment elevation myocardial infarction; BMI, body mass index.

Supplementary Table 8. Baseline characteristics of all NSTEMI patients with different BMI levels in the eICU-CRD cohort.

Characteristics	Overall (n = 1,597)	Normal (18.5-24.9) (n = 376)	Overweight (25.0-29.9) (n = 516)	Obesity I (30.0-34.9) (n = 375)	Severe obesity (≥35.0) (n = 330)	*p* value
**Anthropometric and demographic characteristics**						
Age, years	66.83 ± 12.54	71.53 ± 12.66	67.07 ± 12.66	65.38 ± 11.92	62.76 ± 11.11	<0.001
Sex, n (%)						<0.001
Female	618 (38.70)	161 (42.82)	169 (32.75)	142 (37.87)	146 (44.24)	
Male	979 (61.30)	215 (57.18)	347 (67.25)	233 (62.13)	184 (55.76)	
Weight, kg	87.86 ± 22.77	65.41 ± 9.64	80.45 ± 10.55	94.77 ± 12.30	117.19 ± 21.98	<0.001
Height, cm	170.26 ± 10.48	170.08 ± 10.55	170.89 ± 10.00	170.61 ± 10.52	169.10 ± 11.00	0.018
BMI, kg/m^2	30.23 ± 7.12	22.52 ± 1.70	27.44 ± 1.41	32.44 ± 1.49	40.89 ± 6.12	<0.001
**Comorbidities**						
Hypertension, n (%)						<0.001
No	778 (48.72)	189 (50.27)	260 (50.39)	196 (52.27)	133 (40.30)	
Yes	819 (51.28)	187 (49.73)	256 (49.61)	179 (47.73)	197 (59.70)	
Diabetes mellitus, n (%)						<0.001
No	1,138 (71.26)	277 (73.67)	391 (75.78)	263 (70.13)	207 (62.73)	
Yes	459 (28.74)	99 (26.33)	125 (24.22)	112 (29.87)	123 (37.27)	
Hyperlipidemia, n (%)						<0.001
No	1,392 (87.16)	342 (90.96)	451 (87.40)	329 (87.73)	270 (81.82)	
Yes	205 (12.84)	34 (9.04)	65 (12.60)	46 (12.27)	60 (18.18)	
Stroke, n (%)						<0.001
No	1,544 (96.68)	369 (98.14)	504 (97.67)	365 (97.33)	306 (92.73)	
Yes	53 (3.32)	7 (1.86)	12 (2.33)	10 (2.67)	24 (7.27)	
Atrial fibrillation, n (%)						0.024

No	1,407 (88.10)	323 (85.90)	469 (90.89)	335 (89.33)	280 (84.85)	
Yes	190 (11.90)	53 (14.10)	47 (9.11)	40 (10.67)	50 (15.15)	
**Laboratory parameters**						
Cardiac troponin T, ng/mL	0.34 (0.15,0.80)	0.36 (0.14,0.82)	0.35 (0.17,0.86)	0.30 (0.14,0.78)	0.34 (0.13,0.74)	0.331
Creatine kinase, U/L	263.56 (160.48,376.85)	260.50 (149.08,377.20)	276.25 (172.49,384.38)	259.92 (154.13,368.85)	237.17 (161.59,370.81)	0.093
Creatine kinase MB, U/L	26.15 (12.40,38.61)	26.38 (12.90,41.64)	26.86 (13.26,38.78)	25.63 (10.75,37.15)	23.83 (11.75,38.51)	0.253
C-reactive protein, mg/L	4.10 (1.50,12.90)	3.50 (1.50,10.90)	4.30 (1.40,13.50)	4.40 (1.60,13.90)	4.80 (1.50,14.60)	0.991
White blood cell, 10^9/L	10.12 (8.00,13.06)	9.90 (7.45,12.60)	10.12 (8.27,12.70)	10.12 (8.02,13.10)	10.35 (8.20,13.80)	<0.001
Albumin, g/L	41.20 (38.70,43.60)	40.30 (38.60,42.70)	42.10 (40.70,44.80)	41.80 (39.80,44.10)	38.30 (35.20,40.90)	<0.001
Creatinine, umol/L	94.59 (76.02,133.48)	96.80 (76.02,152.49)	91.05 (74.26,121.11)	94.59 (77.79,147.63)	98.15 (75.60,131.30)	0.028
Urea nitrogen, mmol/L	6.96 (5.00,10.71)	7.56 (5.71,12.05)	6.78 (4.64,9.73)	6.96 (4.91,11.19)	6.78 (5.00,9.95)	<0.001
LDL-C, mmol/L	1.99 (1.50,2.41)	1.97 (1.44,2.35)	1.99 (1.50,2.40)	1.94 (1.42,2.35)	2.12 (1.66,2.57)	<0.001
HDL-C, mmol/L	0.99 (0.92,1.07)	1.01 (0.93,1.08)	1.00 (0.92,1.07)	0.98 (0.91,1.06)	0.97 (0.90,1.06)	<0.001
**Treatment**						
Ventilator, n (%)						<0.001
No	1,493 (93.49)	360 (95.74)	482 (93.41)	356 (94.93)	295 (89.39)	
Yes	104 (6.51)	16 (4.26)	34 (6.59)	19 (5.07)	35 (10.61)	
Intra-aortic balloon pump, n (%)						<0.001
No	1,475 (92.36)	362 (96.28)	476 (92.25)	349 (93.07)	288 (87.27)	
Yes	122 (7.64)	14 (3.72)	40 (7.75)	26 (6.93)	42 (12.73)	
Percutaneous coronary intervention, n (%)						0.036
No	1,290 (80.78)	300 (79.79)	410 (79.46)	295 (78.67)	285 (86.36)	
Yes	307 (19.22)	76 (20.21)	106 (20.54)	80 (21.33)	45 (13.64)	
Coronary-artery-bypass-grafting, n (%)						0.014
No	1,478 (92.55)	352 (93.62)	463 (89.73)	348 (92.80)	315 (95.45)	

Yes	119 (7.45)	24 (6.38)	53 (10.27)	27 (7.20)	15 (4.55)	
**Medication use**						
Aspirin, n (%)						0.013
No	563 (35.25)	152 (40.43)	179 (34.69)	137 (36.53)	95 (28.79)	
Yes	1,034 (64.75)	224 (59.57)	337 (65.31)	238 (63.47)	235 (71.21)	
Clopidogrel, n (%)						0.037
No	1,051 (65.81)	251 (66.76)	351 (68.02)	254 (67.73)	195 (59.09)	
Yes	546 (34.19)	125 (33.24)	165 (31.98)	121 (32.27)	135 (40.91)	
Statins, n (%)						0.013
No	902 (56.48)	235 (62.50)	291 (56.40)	210 (56.00)	166 (50.30)	
Yes	695 (43.52)	141 (37.50)	225 (43.60)	165 (44.00)	164 (49.70)	
β-Blockers, n (%)						<0.001
No	796 (49.84)	215 (57.18)	253 (49.03)	171 (45.60)	157 (47.58)	
Yes	801 (50.16)	161 (42.82)	263 (50.97)	204 (54.40)	173 (52.42)	
Anticoagulation, n (%)						0.015
No	698 (43.71)	183 (48.67)	227 (43.99)	167 (44.53)	121 (36.67)	
Yes	899 (56.29)	193 (51.33)	289 (56.01)	208 (55.47)	209 (63.33)	
ACEI/ARB, n (%)						0.013
No	1,237 (77.46)	312 (82.98)	384 (74.42)	294 (78.40)	247 (74.85)	
Yes	360 (22.54)	64 (17.02)	132 (25.58)	81 (21.60)	83 (25.15)	
**Outcomes**						
**In-hospital all-cause mortality**						<0.001
Alive	1,508 (94.43)	344 (91.49)	500 (96.90)	366 (97.60)	298 (90.30)	
Death	89 (5.57)	32 (8.51)	16 (3.10)	9 (2.40)	32 (9.70)	

Notes: Continuous variables are presented as mean ± SD if normally distributed, and median (interquartile range) if not normally distributed. Categorical variables are presented as number of

patients (%). LDL-C, low-density lipoprotein cholesterol; HDL-C, high-density lipoprotein cholesterol; ACEI, angiotensin-converting enzyme inhibitor; ARB, angiotensin II receptor blocker; NSTEMI, non-ST-segment elevation myocardial infarction; BMI, body mass index.

Supplementary Table 9. Interaction tests between cohort and exposure for in-hospital and 10-year all-cause mortality.

**Exposure**	***P* for interaction (In-hospital all-cause mortality)**	***P* for interaction (10-year all-cause mortality)**
Severe obesity (BMI ≥ 35 kg/m^2 vs. BMI < 35 kg/m^2)	0.978	0.457
Elevated CRP (CRP ≥ 2 mg/L vs. CRP < 2 mg/L)	0.292	0.372

Notes: *P* values were derived from likelihood ratio tests comparing models with and without the interaction term (cohort × exposure). All models were adjusted for age, sex, hypertension, diabetes mellitus, hyperlipidemia, stroke, and atrial fibrillation (Model 3). For in-hospital mortality, interaction tests included all three cohorts (TAMI, MIMIC-IV, eICU-CRD). For 10-year mortality, interaction tests included two cohorts (TAMI and MIMIC-IV) due to the unavailability of 10-year follow-up data in the eICU-CRD cohort. *P* for interaction > 0.05 indicates no significant heterogeneity across cohorts. BMI, body mass index; CRP, C-reactive protein.

Supplementary Table 10. Formal interaction tests between severe obesity and elevated CRP.

Cohort	Outcome	Multiplicative interaction (95% CI)	*p* value	RERI (95% CI)	AP (95% CI)	S (95% CI)
TAMI	In-hospital	0.61 (0.26,1.46)	0.266	1.10 (-1.31,3.52)	0.29 (-0.34,0.91)	1.63 (0.54,7.86)
TAMI	10-year	0.92 (0.57,1.46)	0.704	0.27 (-0.92,1.47)	0.09 (-0.30,0.48)	1.15 (0.61,2.14)
MIMIC-IV	In-hospital	0.68 (0.30,1.57)	0.366	0.03 (-2.02,2.08)	0.01 (-0.52,0.54)	0.99 (0.48,2.75)
MIMIC-IV	10-year	0.73 (0.39,1.37)	0.324	0.05 (-1.49,1.60)	0.02 (-0.43,0.46)	1.02 (0.53,2.39)
eICU-CRD	In-hospital	0.86 (0.31,2.37)	0.774	0.64 (-2.33,3.60)	0.18 (-0.65,0.99)	0.81 (0.26,2.31)

Notes: Multiplicative interaction was assessed by including a product term (Severe obesity × elevated CRP) in the fully adjusted Cox model (Model 3). A p value < 0.05 indicates significant multiplicative interaction. Additive interaction was evaluated using the relative excess risk due to interaction (RERI), attributable proportion (AP), and synergy index (S). RERI > 0, AP > 0, and S > 1 indicate positive additive interaction, with statistical significance assessed by whether the 95% confidence interval (CI) excludes the null (0 for RERI and AP, 1 for S). RERI, relative excess risk due to interaction; AP, attributable proportion; S, synergy index; CRP, C-reactive protein. CI, confidence interval.

Supplementary Table 11. Interaction tests for severe obesity, elevated CRP, and diabetes for in-hospital and 10-year all-cause mortality.

**Interaction term**	***P* for interaction (In-hospital all-cause mortality)**	***P* for interaction (10-year all-cause mortality)**
Severe obesity × Diabetes mellitus	0.198	0.252
Elevated CRP × Diabetes mellitus	0.394	0.441
Severe obesity × Elevated CRP	0.178	0.169

Notes: P values were derived from likelihood ratio tests comparing models with and without the interaction term using pooled data from the three cohorts (TAMI, MIMIC-IV, and eICU-CRD). All models were adjusted for age, sex, hypertension, hyperlipidemia, stroke, atrial fibrillation, and the other main effects as appropriate (e.g., models testing BMI × DM were adjusted for CRP, and models testing CRP × DM were adjusted for BMI). P for interaction > 0.05 indicates no significant effect modification. BMI, body mass index; CRP, C-reactive protein; DM, diabetes mellitus.

Supplementary Table 12. Evaluation of predictive models for all-cause mortality.

	**C-index (95% CI)**	***p* value**	**NRI (95% CI, %)**	***p* value**	**IDI (95% CI, %)**	***p* value**
**TAMI cohort**						
**In-hospital all-cause mortality**						
Conventional model	0.764 (0.744,0.783)		Reference		Reference	
Conventional model + CRP	0.768 (0.749,0.788)	0.021	24.24 (9.52,38.97)	0.001	0.61 (0.03,1.18)	0.037
**10-year all-cause mortality**						
Conventional model	0.796 (0.778,0.815)		Reference		Reference	
Conventional model + CRP	0.801 (0.783,0.820)	<0.001	21.08 (13.06,29.10)	<0.001	0.29 (0.08,0.49)	0.006
**MIMIC-IV cohort**						
**In-hospital all-cause mortality**						
Conventional model	0.600 (0.563,0.638)		Reference		Reference	
Conventional model + CRP	0.612 (0.575,0.650)	<0.001	26.18 (7.21,45.14)	0.007	4.01 (1.28,6.71)	0.004
**10-year all-cause mortality**						
Conventional model	0.710 (0.675,0.746)		Reference		Reference	
Conventional model + CRP	0.733 (0.700,0.767)	0.007	39.59 (27.35,51.28)	<0.001	1.21 (0.61,1.80)	<0.001
**eICU-CRD cohort**						
**In-hospital all-cause mortality**						
Conventional model	0.781 (0.727,0.835)		Reference		Reference	
Conventional model + CRP	0.791 (0.738,0.844)	0.013	38.96 (20.10,57.83)	<0.001	1.25 (0.33,2.18)	0.008

Notes: Conventional model includes BMI (category), age, sex, hypertension, diabetes mellitus, hyperlipidemia, stroke, atrial fibrillation. C-index, NRI, and IDI are measures of model discrimination and reclassification; higher values indicate better predictive performance. Statistical significance for NRI and IDI was assessed using bootstrap methods (1000 resamples). BMI, body mass index; CRP, C-reactive protein; NRI, net reclassification improvement; IDI, integrated discrimination index.

Supplementary Table 13. Fine-Gray competing risk regression for BMI and 10-year all-cause mortality treating in-hospital death as a competing event.

BMI	Model 1	*p* value	Model 2	*p* value	Model 3	*p* value
	SHR (95% CI)		SHR (95% CI)		SHR (95% CI)	
**TAMI cohort**						
**10-year all-cause mortality**						
BMI (continuous)	1.04 (1.02,1.06)	<0.001	1.04 (1.02,1.06)	<0.001	1.02 (1.00,1.04)	0.017
BMI (four categories)						
Normal (18.5-24.9)	Reference		Reference		Reference	
Overweight (25.0-29.9)	0.57 (0.46,0.70)	<0.001	0.70 (0.56,0.86)	<0.001	0.61 (0.49,0.75)	<0.001
Obesity I (30.0-34.9)	0.58 (0.43,0.78)	<0.001	0.74 (0.54,0.99)	0.048	0.59 (0.44,0.81)	0.001
Severe obesity (≥ 35.0)	1.49 (1.11,2.00)	0.008	1.73 (1.28,2.33)	<0.001	1.35 (1.01,1.81)	0.048
BMI (two categories)						
Non-severe obesity (< 35.0)	Reference		Reference		Reference	
Severe obesity (≥ 35.0)	1.94 (1.47,2.85)	<0.001	2.08 (1.57,2.76)	<0.001	1.80 (1.36,2.37)	<0.001
**MIMIC-IV cohort**						
**10-year all-cause mortality**						
BMI (continuous)	1.01 (0.99,1.03)	0.109	1.01 (0.99,1.03)	0.144	1.01 (0.99,1.03)	0.269
BMI (four categories)						
Normal (18.5-24.9)	Reference		Reference		Reference	
Overweight (25.0-29.9)	0.77 (0.54,1.08)	0.127	0.70 (0.50,0.98)	0.041	0.68 (0.48,0.96)	0.028
Obesity I (30.0-34.9)	0.79 (0.54,1.14)	0.206	0.74 (0.51,1.07)	0.114	0.73 (0.49,1.07)	0.104
Severe obesity (≥ 35.0)	1.31 (0.95,1.82)	0.101	1.44 (1.04,1.99)	0.031	1.43 (1.01,2.04)	0.046
BMI (two categories)						
Non-severe obesity (< 35.0)	Reference		Reference		Reference	
Severe obesity (≥ 35.0)	1.54 (1.15,2.08)	<0.001	1.71 (1.28,2.29)	<0.001	1.79 (1.31,2.44)	<0.001

Notes: Fine-Gray competing risk regression models were used, treating in-hospital death as a competing event for 10-year all-cause mortality. Model 1, unadjusted; Model 2, adjusted for age, sex; Model 3, adjusted for model 2 plus hypertension, diabetes mellitus, hyperlipidemia, stroke, atrial fibrillation. BMI, body mass index; SHR, sub-distribution hazard ratio; CI, confidence interval.

Supplementary Table 14. Multivariate-adjusted HR (95% CI) of BMI and 10-year all-cause mortality after excluding patients who died within one year.

BMI	Event/Total (%)	Model 1 HR (95% CI)	*p* value	Model 2 HR (95% CI)	*p* value	Model 3 HR (95% CI)	*p* value
**TAMI cohort**							
**10-year all-cause mortality**							
BMI (continuous)	402/4,777 (8.42)	1.04 (1.02,1.06)	<0.001	1.05 (1.03,1.07)	<0.001	1.04 (1.02,1.06)	<0.001
BMI (four categories)							
Normal (18.5-24.9)	99/1,225 (8.08)	Reference		Reference		Reference	
Overweight (25.0-29.9)	131/1,967 (6.66)	0.62 (0.50,0.78)	<0.001	0.75 (0.60,0.95)	0.015	0.65 (0.52,0.82)	<0.001
Obesity I (30.0-34.9)	86/1,058 (8.13)	0.62 (0.44,0.84)	0.003	0.79 (0.57,1.09)	0.153	0.63 (0.46,0.88)	0.007
Severe obesity (≥ 35.0)	86/527 (16.32)	1.76 (1.29,2.41)	<0.001	2.18 (1.59,2.98)	<0.001	1.76 (1.28,2.42)	<0.001
BMI (two categories)							
Non-severe obesity (< 35.0)	316/4,250 (7.44)	Reference		Reference		Reference	
Severe obesity (≥ 35.0)	86/527 (16.32)	2.33 (1.74,3.12)	<0.001	2.54 (1.89,3.41)	<0.001	2.28 (1.70,3.06)	<0.001
**MIMIC-IV cohort**							
**10-year all-cause mortality**							
BMI (continuous)	83/1,031 (8.05)	1.03 (0.99,1.05)	0.071	1.03 (1.00,1.06)	0.052	1.03 (1.01,1.06)	0.042
BMI (four categories)							
Normal (18.5-24.9)	28/350 (8.00)	Reference		Reference		Reference	
Overweight (25.0-29.9)	19/273 (6.96)	1.14 (0.64,2.05)	0.655	1.07 (0.59,1.94)	0.819	1.01 (0.56,1.83)	0.977
Obesity I (30.0-34.9)	10/238 (4.20)	0.67 (0.32,1.38)	0.277	0.60 (0.29,1.25)	0.173	0.57 (0.27,1.20)	0.139
Severe obesity (≥ 35.0)	26/170 (15.29)	1.72 (1.01,2.93)	0.048	1.79 (1.05,3.07)	0.033	2.04 (1.14,3.62)	0.016
BMI (two categories)							
Non-severe obesity (< 35.0)	57/861 (6.62)	Reference		Reference		Reference	
Severe obesity (≥ 35.0)	26/170 (15.29)	1.79 (1.13,2.85)	0.014	1.96 (1.23,3.12)	0.005	2.28 (1.37,3.79)	0.001

Notes: This sensitivity analysis excluded patients who died within the first year of follow-up to assess the potential impact of reverse causation. Model 1 was unadjusted. Model 2 was adjusted for age and sex. Model 3 was further adjusted for hypertension, diabetes mellitus, hyperlipidemia, stroke, and atrial fibrillation. BMI, body mass index; HR, hazard ratio; CI, confidence interval.

Supplementary Table 15. Multivariate-adjusted HR (95% CI) of BMI categories for all-cause mortality after additional adjustment for BMI as a continuous variable.

**BMI**	**Event/Total (%)**	**Model 1**	***p* value**	**Model 2**	***p* value**	**Model 3**	***p* value**
		**HR (95% CI)**		**HR (95% CI)**		**HR (95% CI)**	
**TAMI cohort**							
**10-year all-cause mortality**							
BMI (four categories)							
Normal (18.5-24.9)	283/1,912 (14.80)	Reference		Reference		Reference	
Overweight (25.0-29.9)	181/2,060 (8.79)	0.52 (0.44,0.63)	<0.001	0.59 (0.48,0.71)	<0.001	0.46 (0.37,0.57)	<0.001
Obesity I (30.0-35.0)	81/715 (11.33)	0.67 (0.52,0.85)	0.001	0.74 (0.58,0.95)	0.017	0.44 (0.31,0.63)	0.017
Severe obesity (≥ 35.0)	90/323 (27.86)	1.66 (1.29,2.12)	<0.001	1.68 (1.31,2.15)	<0.001	1.65 (1.37,2.14)	<0.001
BMI (two categories)							
Non-severe obesity (< 35.0)	545/4,687 (11.63)	Reference		Reference		Reference	
Severe obesity (≥ 35.0)	90/323 (27.86)	2.29 (1.81,2.88)	<0.001	2.18 (1.73,2.76)	<0.001	2.43 (1.70,3.47)	<0.001
**In-hospital all-cause mortality**							
BMI (four categories)							
Normal (18.5-24.9)	79/1,912 (4.13)	Reference		Reference		Reference	
Overweight (25.0-29.9)	32/2,060 (1.55)	0.41 (0.27,0.61)	<0.001	0.50 (0.33,0.76)	0.001	0.36 (0.23,0.57)	<0.001
Obesity I (30.0-35.0)	12/715 (1.68)	0.39 (0.21,0.71)	0.002	0.45 (0.24,0.83)	0.011	0.22 (0.11,0.47)	<0.001
Severe obesity (≥ 35.0)	27/323 (8.36)	1.62 (1.04,2.51)	0.032	1.69 (1.08,2.65)	0.022	1.57 (1.04,2.45)	0.028
BMI (two categories)							
Non-severe obesity (< 35.0)	125/4,687 (2.67)	Reference		Reference		Reference	
Severe obesity (≥ 35.0)	27/323 (8.36)	2.48 (1.63,3.78)	<0.001	2.37 (1.55,3.61)	<0.001	2.59 (1.32,5.08)	<0.001
**MIMIC-IV cohort**							
**10-year all-cause mortality**							

BMI (four categories)							
Normal (18.5-24.9)	99/421 (23.52)	Reference		Reference		Reference	
Overweight (25.0-30.0)	46/300 (15.33)	0.66 (0.47,0.94)	0.021	0.60 (0.42,0.85)	0.004	0.66 (0.45,0.98)	0.041
Obesity I (30.0-34.9)	42/270 (15.56)	0.67 (0.47,0.96)	0.029	0.61 (0.43,0.88)	0.008	0.66 (0.45,0.97)	0.044
Severe obesity (≥ 35.0)	73/217 (33.64)	1.36 (1.01,1.84)	0.046	1.52 (1.14,2.15)	0.006	2.26 (1.11,4.61)	0.025
BMI (two categories)							
Non-severe obesity (< 35.0)	187/991 (18.87)	Reference		Reference		Reference	
Severe obesity (≥ 35.0)	73/217 (33.64)	1.68 (1.28,2.20)	<0.001	2.04 (1.53,2.73)	<0.001	2.55 (1.21,4.72)	<0.001
**In-hospital all-cause mortality**							
BMI (four categories)							
Normal (18.5-24.9)	38/421 (9.03)	Reference		Reference		Reference	
Overweight (25.0-29.9)	9/300 (3.00)	0.42 (0.21,0.86)	0.017	0.44 (0.21,0.89)	0.022	0.48 (0.25,0.93)	0.029
Obesity I (30.0-35.0)	6/270 (2.22)	0.26 (0.12,0.59)	0.001	0.25 (0.11,0.57)	<0.001	0.31 (0.14,0.68)	0.004
Severe obesity (≥ 35.0)	35/217 (16.13)	1.58 (1.01,2.45)	0.045	1.60 (1.02,2.52)	0.039	1.59 (1.09,2.78)	0.034
BMI (two categories)							
Non-severe obesity (< 35.0)	53/991 (5.35)	Reference		Reference		Reference	
Severe obesity (≥ 35.0)	35/217 (16.13)	2.69 (1.82,3.98)	<0.001	2.66 (1.78,3.98)	<0.001	2.92 (2.05,4.51)	<0.001
**eICU-CRD cohort**							
**In-hospital all-cause mortality**							
BMI (four categories)							
Normal (18.5-24.9)	32/376 (8.51)	Reference		Reference		Reference	
Overweight (25.0-29.9)	16/516 (3.10)	0.38 (0.21,0.70)	0.002	0.50 (0.27,0.93)	0.027	0.38 (0.20,0.73)	0.003
Obesity I (30.0-34.9)	9/375 (2.40)	0.25 (0.12,0.53)	<0.001	0.34 (0.16,0.72)	0.005	0.19 (0.08,0.45)	<0.001
Severe obesity (≥ 35.0)	32/330 (9.70)	1.16 (0.71,1.90)	0.545	1.80 (1.04,3.14)	0.038	1.86 (1.07,3.21)	0.029
**BMI (two categories)**							

Non-severe obesity (< 35.0)	57/1,267 (4.50)	Reference		Reference		Reference	
Severe obesity (≥ 35.0)	32/330 (9.70)	2.24 (1.45,3.46)	<0.001	3.01 (1.86,4.86)	<0.001	2.95 (1.39,6.28)	0.005

Notes: Model 1 was unadjusted. Model 2 was adjusted for age, sex, hypertension, diabetes mellitus, hyperlipidemia, stroke, and atrial fibrillation (equivalent to the fully adjusted model in the primary analysis). Model 3 was further adjusted for BMI as a continuous variable. BMI, body mass index; HR, hazard ratio; CI, confidence interval.

Supplementary Table 16. E-values for unmeasured confounding in the association between BMI categories and all-cause mortality.

Cohort	Outcome	BMI category	HR (95% CI)	Effect direction	E-value
TAMI	10-year	Overweight (25.0-29.9)	0.59 (0.48–0.71)	Protective	2.24
		Obesity I (30.0-34.9)	0.74 (0.58–0.95)	Protective	1.77
		Severe obesity (≥ 35.0)	1.68 (1.31–2.15)	Risk	2.22
	In-hospital	Overweight (25.0-29.9)	0.50 (0.33–0.76)	Protective	2.61
		Obesity I (30.0-34.9)	0.45 (0.24–0.83)	Protective	2.86
		Severe obesity (≥ 35.0)	1.69 (1.08–2.65)	Risk	2.23
MIMIC-IV	10-year	Overweight (25.0-29.9)	0.60 (0.42–0.85)	Protective	2.21
		Obesity I (30.0-34.9)	0.61 (0.43–0.88)	Protective	2.16
		Severe obesity (≥ 35.0)	1.56 (1.14–2.15)	Risk	2.06
	In-hospital	Overweight (25.0-29.9)	0.44 (0.21–0.89)	Protective	2.91
		Obesity I (30.0-34.9)	0.25 (0.11–0.57)	Protective	4.56
		Severe obesity (≥ 35.0)	1.60 (1.02–2.52)	Risk	2.11
eICU-CRD	In-hospital	Overweight (25.0-29.9)	0.50 (0.27–0.93)	Protective	2.61
		Obesity I (30.0-34.9)	0.34 (0.16–0.72)	Protective	3.61
		Severe obesity (≥ 35.0)	1.80 (1.04–3.14)	Risk	2.37

Notes: E-values quantify the minimum strength of association (on the risk ratio scale) that an unmeasured confounder would need to have with both the exposure (BMI category) and the outcome (all-cause mortality) to fully explain the observed association, after accounting for measured covariates. Larger E-values indicate greater robustness to unmeasured confounding. For protective effects (HR < 1), the HR was inverted to a risk ratio (RR = 1/HR) before E-value calculation. CI, confidence interval; HR, hazard ratio; RR, risk ratio; BMI, body mass index.

Supplementary Table 17. Sensitivity analysis using Chinese obesity classification criteria for BMI and all-cause mortality in the TAMI cohort.

BMI	Event/Total (%)	Model 1	*p* value	Model 2	*p* value	Model 3	*p* value
		HR (95% CI)		HR (95% CI)		HR (95% CI)	
**TAMI cohort**							
**10-year all-cause mortality**							
BMI (continuous)	635/5,010 (12.67)	1.04 (1.02,1.05)	<0.001	1.05 (1.04,1.07)	<0.001	1.04 (1.02,1.05)	<0.001
BMI (four categories)							
Normal (18.5-23.9)	193/1,319 (14.63)	Reference		Reference		Reference	
Overweight (24.0-27.9)	173/2,009 (8.61)	0.48 (0.39,0.59)	<0.001	0.59 (0.48,0.72)	<0.001	0.61 (0.50,0.75)	<0.001
Obesity I (28.0-32.5)	120/1,092 (10.99)	0.62 (0.49,0.78)	<0.001	0.73 (0.58,0.92)	0.007	0.70 (0.56,0.88)	0.003
Severe obesity (≥32.5)	149/590 (25.25)	1.61 (1.30,1.99)	<0.001	2.23 (1.80,2.76)	<0.001	1.83 (1.47,2.27)	<0.001
BMI (two categories)							
Non-severe obesity (< 32.5)	486/4,420 (11.00)	Reference		Reference		Reference	
Severe obesity (≥32.5)	149/590 (25.25)	2.46 (2.05,2.96)	<0.001	2.98 (2.48,3.58)	<0.001	2.44 (2.02,2.94)	<0.001
**In-hospital all-cause mortality**							
BMI (continuous)	150/5010 (2.99)	1.03 (1.00,1.06)	0.031	1.04 (1.01,1.07)	0.015	1.03 (0.99,1.06)	0.057
BMI (four categories)							
Normal (18.5-23.9)	55/1319 (4.17)	Reference		Reference		Reference	
Overweight (24.0-27.9)	29/2009 (1.44)	0.38 (0.24,0.60)	<0.001	0.47 (0.30,0.74)	0.001	0.55 (0.35,0.87)	0.011
Obesity I (28.0-32.5)	18/1092 (1.65)	0.40 (0.23,0.68)	<0.001	0.44 (0.26,0.75)	0.002	0.50 (0.29,0.87)	0.013
Severe obesity (≥32.5)	48/590 (8.14)	1.69 (1.14,2.49)	0.008	1.94 (1.31,2.86)	<0.001	1.74 (1.15,2.62)	0.008
**BMI (two categories)**							
Non-severe obesity (< 32.5)	102/4,420 (2.31)	Reference		Reference		Reference	
Severe obesity (≥32.5)	48/590 (8.14)	2.89 (2.05,4.09)	<0.001	3.00 (2.12,4.23)	<0.001	2.46 (1.71,3.54)	<0.001

Notes: This sensitivity analysis applied the Chinese obesity classification criteria to the TAMI cohort, with BMI categories defined as normal (18.5–23.9 kg/m²), overweight (24.0–27.9 kg/m²), obesity I (28.0–32.5 kg/m²), and obesity II/III (≥32.5 kg/m²). Model 1 was unadjusted. Model 2 was adjusted for age and sex. Model 3 was further adjusted for hypertension, diabetes mellitus,

hyperlipidemia, stroke, and atrial fibrillation. BMI, body mass index; HR, hazard ratio; CI, confidence interval.

Supplementary Table 18. Multivariate-adjusted HR (95% CI) of BMI combined with CRP and all-cause mortality after excluding patients with missing CRP.

Characteristics	Event/Total (%)	Model 1	*p* value	Model 2	*p* value	Model 3	*p* value
		HR (95% CI)		HR (95% CI)		HR (95% CI)	
**TAMI cohort**							
**In-hospital all-cause mortality**							
BMI < 35kg/m^2 and CRP < 2mg/L	37/1,940 (1.91)	Reference		Reference		Reference	
BMI < 35kg/m^2 and CRP ≥ 2mg/L	78/2,467 (3.16)	1.59 (1.07,2.35)	0.021	1.56 (1.06,2.31)	0.026	1.53 (1.03,2.26)	0.035
BMI ≥ 35kg/m^2 and CRP < 2mg/L	11/166 (6.63)	2.78 (1.42,5.46)	0.003	2.61 (1.33,5.12)	0.005	2.58 (1.31,5.09)	0.006
BMI ≥ 35kg/m^2 and CRP ≥ 2mg/L	14/135 (10.37)	4.10 (2.21,7.60)	<0.001	3.91 (2.10,7.26)	<0.001	3.81 (2.04,7.13)	<0.001
**10-year all-cause mortality**							
BMI < 35kg/m^2 and CRP < 2mg/L	180/1,940 (9.28)	Reference		Reference		Reference	
BMI < 35kg/m^2 and CRP ≥ 2mg/L	334/2,467 (13.54)	1.45 (1.21,1.73)	<0.001	1.39 (1.16,1.66)	<0.001	1.39 (1.16,1.67)	<0.001
BMI ≥ 35kg/m^2 and CRP < 2mg/L	36/166 (21.69)	2.49 (1.74,3.57)	<0.001	2.79 (1.95,3.99)	<0.001	2.48 (1.74,3.56)	<0.001
BMI ≥ 35kg/m^2 and CRP ≥ 2mg/L	51/135 (37.78)	3.48 (2.49,4.85)	<0.001	3.20 (2.29,4.49)	<0.001	2.98 (2.13,4.16)	<0.001
**MIMIC-IV cohort**							
**In-hospital all-cause mortality**							
BMI < 35kg/m^2 and CRP < 2mg/L	9/341 (2.64)	Reference		Reference		Reference	
BMI < 35kg/m^2 and CRP ≥ 2mg/L	28/461 (6.07)	2.52 (1.16,5.48)	0.021	2.55 (1.17,5.57)	0.019	2.45 (1.12,5.37)	0.025
BMI ≥ 35kg/m^2 and CRP < 2mg/L	9/60 (15.00)	3.16 (1.25,8.03)	0.015	3.25 (1.28,8.25)	0.013	2.98 (1.15,7.69)	0.024
BMI ≥ 35kg/m^2 and CRP ≥ 2mg/L	17/107 (15.89)	3.92 (1.75,8.79)	<0.001	4.12 (1.84,9.27)	<0.001	3.78 (1.67,8.53)	<0.001
**10-year all-cause mortality**							
BMI < 35kg/m^2 and CRP < 2mg/L	37/341 (10.85)	Reference		Reference		Reference	
BMI < 35kg/m^2 and CRP ≥ 2mg/L	111/461 (24.08)	1.99 (1.37,2.89)	<0.001	1.81 (1.24,2.63)	0.002	1.90 (1.30,2.77)	<0.001
BMI ≥ 35kg/m^2 and CRP < 2mg/L	16/60 (26.67)	2.31 (1.29,4.16)	0.009	2.39 (1.33,4.31)	0.004	2.43 (1.33,4.44)	0.004
BMI ≥ 35kg/m^2 and CRP ≥ 2mg/L	43/107 (40.19)	3.21 (2.07,4.99)	<0.001	3.37 (2.17,5.24)	<0.001	3.56 (2.25,5.64)	<0.001

**eICU-CRD cohort**							
**In-hospital all-cause mortality**							
BMI < 35.0kg/m^2 and CRP < 2mg/L	9/389 (2.31)	Reference		Reference		Reference	
BMI < 35.0kg/m^2 and CRP ≥ 2mg/L	36/536 (6.72)	2.92 (1.40,6.06)	0.004	2.58 (1.24,5.39)	0.011	2.40 (1.14,5.04)	0.021
BMI ≥ 35.0kg/m^2 and CRP < 2mg/L	7/128 (5.47)	2.56 (0.95,6.91)	0.063	3.30 (1.21,8.96)	0.019	3.14 (1.14,8.67)	0.027
BMI ≥ 35.0kg/m^2 and CRP ≥ 2mg/L	6/177 (9.04)	4.07 (1.79,9.25)	<0.001	4.56 (2.00,10.39)	<0.001	4.51 (1.94,10.48)	<0.001

Notes: This sensitivity analysis excluded patients with missing CRP values and re-analyzed the joint effects of BMI and CRP using complete-case analysis. The results were consistent with the primary analysis that used multiple imputation, confirming the robustness of the findings. Model 1 was unadjusted. Model 2 was adjusted for age and sex. Model 3 was further adjusted for hypertension, diabetes mellitus, hyperlipidemia, stroke, and atrial fibrillation. BMI, body mass index; CRP, C-reactive protein; HR, hazard ratio; CI, confidence interval.

Supplementary Table 19. Multivariable-adjusted HR (95% CI) of BMI and all-cause mortality in the three cohorts (additional adjustment for PCI, CABG, cTnT and albumin).

BMI	Event/Total (%)	Model 1	*p* value	Model 2	*p* value	Model 3	*p* value
		HR (95% CI)		HR (95% CI)		HR (95% CI)	
**TAMI cohort**							
**In-hospital all-cause mortality**							
BMI (continuous)	150/5010 (2.99)	1.04 (1.01,1.07)	0.011	1.04 (1.01,1.07)	0.016	1.04 (1.01,1.07)	0.008
BMI (four categories)							
Normal (18.5-24.9)	79/1,912 (4.13)	Reference		Reference		Reference	
Overweight (25.0-29.9)	32/2,060 (1.55)	0.51 (0.34,0.78)	0.002	0.49 (0.32,0.75)	<0.001	0.52 (0.34,0.79)	0.002
Obesity I (30.0-34.9)	12/715 (1.68)	0.46 (0.25,0.85)	0.002	0.43 (0.23,0.80)	0.008	0.43 (0.23,0.80)	0.007
Severe obesity (≥ 35.0)	27/323 (8.36)	1.73 (1.11,2.71)	0.017	1.59 (1.02,2.50)	0.044	1.68 (1.06,2.65)	0.026
BMI (two categories)							
Non-severe obesity (< 35.0)	125/4,687 (2.67)	Reference		Reference		Reference	
Severe obesity (≥ 35.0)	27/323 (8.36)	2.40 (1.57,3.66)	<0.001	2.27 (1.49,3.47)	<0.001	2.39 (1.56,3.66)	<0.001
**10-year all-cause mortality**							
BMI (continuous)	635/5,010 (12.67)	1.04 (1.03,1.06)	<0.001	1.04 (1.02,1.05)	<0.001	1.04 (1.02,1.06)	<0.001
BMI (four categories)							
Normal (18.5-24.9)	283/1,912 (14.80)	Reference		Reference		Reference	
Overweight (25.0-29.9)	181/2,060 (8.79)	0.59 (0.49,0.71)	<0.001	0.57 (0.48,0.65)	<0.001	0.58 (0.48,0.70)	<0.001
Obesity I (30.0-34.9)	81/715 (11.33)	0.75 (0.58,0.96)	0.023	0.73 (0.57,0.94)	0.014	0.72 (0.56,0.93)	0.011
Severe obesity (≥ 35.0)	90/323 (27.86)	1.68 (1.26,2.16)	<0.001	1.68 (1.27,2.15)	<0.001	1.70 (1.29,2.24)	<0.001
BMI (two categories)							
Non-severe obesity (< 35.0)	545/4,687 (11.63)	Reference		Reference		Reference	
Severe obesity (≥ 35.0)	90/323 (27.86)	2.16 (1.71,2.78)	<0.001	2.16 (1.69,2.85)	<0.001	2.19 (1.68,2.82)	<0.001

**MIMIC-IV cohort**							
**In-hospital all-cause mortality**							
BMI (continuous)	88/1,208 (7.28)	1.03 (1.01,1.06)	0.018	1.03 (1.01,1.06)	0.018	1.03 (1.01,1.06)	0.016
BMI (four categories)							
Normal (18.5-24.9)	38/421 (9.03)	Reference		Reference		Reference	
Overweight (25.0-29.9)	9/300 (3.00)	0.46 (0.22,0.94)	0.032	0.46 (0.22,0.94)	0.032	0.47 (0.23,0.97)	0.042
Obesity I (30.0-34.9)	6/270 (2.22)	0.26 (0.11,0.59)	0.001	0.26 (0.11,0.59)	0.001	0.26 (0.12,0.60)	0.002
Severe obesity (≥ 35.0)	35/217 (16.13)	1.58 (1.01,2.48)	0.047	1.59 (1.01,2.49)	0.046	1.58 (1.01,2.48)	0.046
BMI (two categories)							
Non-severe obesity (< 35.0)	53/991 (5.35)	Reference		Reference		Reference	
Severe obesity (≥ 35.0)	35/217 (16.13)	2.55 (1.70,3.82)	<0.001	2.56 (1.70,3.84)	<0.001	2.50 (1.66,3.74)	<0.001
**10-year all-cause mortality**							
BMI (continuous)	260/1,208 (21.52)	1.01 (0.99,1.03)	0.232	1.01 (0.99,1.03)	0.241	1.01 (0.99,1.03)	0.191
BMI (four categories)							
Normal (18.5-24.9)	99/421 (23.52)	Reference		Reference		Reference	
Overweight (25.0-29.9)	46/300 (15.33)	0.60 (0.42,0.86)	0.005	0.60 (0.42,0.85)	0.004	0.61 (0.43,0.86)	0.005
Obesity I (30.0-34.9)	42/270 (15.56)	0.60 (0.42,0.86)	0.006	0.60 (0.42,0.86)	0.005	0.62 (0.43,0.89)	0.009
Severe obesity (≥ 35.0)	73/217 (33.64)	1.44 (1.04,1.98)	0.027	1.43 (1.04,1.98)	0.028	1.43 (1.04,1.97)	0.027
BMI (two categories)							
Non-severe obesity (< 35.0)	187/991 (18.87)	Reference		Reference		Reference	
Severe obesity (≥ 35.0)	73/217 (33.64)	1.89 (1.41,2.53)	<0.001	1.89 (1.41,2.53)	<0.001	1.86 (1.39,2.49)	<0.001
**eICU-CRD cohort**							
**In-hospital all-cause mortality**							
BMI (continuous)	89/1,597 (5.57)	1.05 (1.02,1.08)	<0.001	1.05 (1.03,1.08)	<0.001	1.05 (1.03,1.08)	<0.001
BMI (four categories)							

Normal (18.5-24.9)	32/376 (8.51)	Reference		Reference		Reference	
Overweight (25.0-29.9)	16/516 (3.10)	0.49 (0.27,0.91)	0.023	0.47 (0.26,0.87)	0.016	0.49 (0.27,0.90)	0.021
Obesity I (30.0-34.9)	9/375 (2.40)	0.33 (0.15,0.70)	0.004	0.33 (0.16,0.71)	0.005	0.35 (0.16,0.74)	0.006
Severe obesity (≥ 35.0)	32/330 (9.70)	1.87 (1.07,3.24)	0.027	1.95 (1.12,3.38)	0.018	2.03 (1.17,3.53)	0.012
BMI (two categories)							
Non-severe obesity (< 35.0)	57/1,267 (4.50)	Reference		Reference		Reference	
Severe obesity (≥ 35.0)	32/330 (9.70)	3.15 (1.95,5.01)	<0.001	3.30 (2.04,5.37)	<0.001	3.38 (2.09,5.48)	<0.001

Notes: Model 1: adjusted for age, sex, hypertension, diabetes mellitus, hyperlipidemia, stroke, atrial fibrillation, PCI and CABG; Model 2: further adjusted for cTnT; Model 3: further adjusted for albumin. PCI, percutaneous coronary intervention; CABG, coronary-artery-bypass-grafting; cTnT, cardiac troponin T. BMI, body mass index; HR, hazard ratio; CI, confidence interval; TAMI, Tianjin Inpatient Acute Myocardial Infarction; MIMIC-IV, Medical Information Mart for Intensive Care IV; eICU-CRD, eICU Collaborative Research Database.
